# A cell wall reference profile for *Miscanthus* bioenergy crops highlights compositional and structural variations associated with development and organ origin

**DOI:** 10.1111/nph.14306

**Published:** 2016-11-15

**Authors:** Ricardo M. F. da Costa, Sivakumar Pattathil, Utku Avci, Scott J. Lee, Samuel P. Hazen, Ana Winters, Michael G. Hahn, Maurice Bosch

**Affiliations:** ^1^ Institute of BiologicalEnvironmental and Rural SciencesAberystwyth UniversityPlas GogerddanAberystwythCeredigionSY23 3EEUK; ^2^ Complex Carbohydrate Research CenterThe University of Georgia315 Riverbend RoadAthensGA30602USA; ^3^ US Department of Energy Bioenergy Science CenterOak Ridge National LaboratoryOak RidgeTN37831USA; ^4^ Biology DepartmentUniversity of MassachusettsAmherstMA01003USA

**Keywords:** bioenergy, biomass, carbohydrate, cell wall, glycan, lignocellulose, miscanthus, recalcitrance

## Abstract

- *Miscanthus* spp. are promising lignocellulosic energy crops, but cell wall recalcitrance to deconstruction still hinders their widespread use as bioenergy and biomaterial feedstocks. Identification of cell wall characteristics desirable for biorefining applications is crucial for lignocellulosic biomass improvement. However, the task of scoring biomass quality is often complicated by the lack of a reference for a given feedstock.
- A multidimensional cell wall analysis was performed to generate a reference profile for leaf and stem biomass from several miscanthus genotypes harvested at three developmentally distinct time points. A comprehensive suite of 155 monoclonal antibodies was used to monitor changes in distribution, structure and extractability of noncellulosic cell wall matrix glycans.
- Glycan microarrays complemented with immunohistochemistry elucidated the nature of compositional variation, and *in situ* distribution of carbohydrate epitopes. Key observations demonstrated that there are crucial differences in miscanthus cell wall glycomes, which may impact biomass amenability to deconstruction.
- For the first time, variations in miscanthus cell wall glycan components were comprehensively characterized across different harvests, organs and genotypes, to generate a representative reference profile for miscanthus cell wall biomass. Ultimately, this portrait of the miscanthus cell wall will help to steer breeding and genetic engineering strategies for the development of superior energy crops.

## Introduction

Global hydrocarbon availability has reportedly been increased thanks to modern hydraulic fracturing (Norris *et al*., 2016). However, studies have reported that these extraction technologies employ toxic, allergenic, mutagenic and carcinogenic chemical additives, and cause the surfacing of radioactive materials (Lechtenböhmer *et al*., 2011). At a time when sustainability is crucial, ‘greener’ solutions for the production of fuel and other bioproducts may be found in lignocellulosic biomass. Dedicated second‐generation bioenergy crops can provide such biomass, and the *Miscanthus* genus contains species with high potential as sustainable biomass providers (Carroll & Somerville, 2009).

Considering their high biomass yields, perenniality, C_4_ carbon fixation, potential for soil carbon sequestration, reduced soil erosion and low fertilizer requirement (Clifton‐Brown *et al*., 2013; van der Weijde *et al*., 2013), the most relevant miscanthus varieties include *M*. *sinensis*,* M*. *sacchariflorus* and *M*. × *giganteus* (Heaton *et al*., 2008; Dwiyanti *et al*., 2013; Liu *et al*., 2013). In the UK, miscanthus is planted during spring, and once established can be harvested annually for up to 15 yr (DEFRA, 2007). New shoots typically emerge by midspring and grow rapidly in the following months, reaching several metres in height by midsummer, depending on genotype. Autumn frosts trigger senescence, and senescing miscanthus remobilizes nutrients from above‐ground organs to rhizomes. For this reason, only completely senesced plants are usually harvested, thus ensuring crop regrowth in the subsequent season.

Despite numerous potential biorefining applications, lignocellulosic biomass still remains largely untapped as a consequence of cell wall recalcitrance, which refers to the resistance of cell walls to deconstruction (Himmel *et al*., 2007), dictated by relative abundances and interactions between cell wall components (Pauly & Keegstra, 2010). Accordingly, for the aim of efficient utilization of cell wall biomass as a renewable source of useful molecules, it is vital to further our knowledge regarding how walls are assembled, in terms of both composition and structure.

Commelinoid monocots, which include grasses, contain cell walls that are distinct from those found in other plant *taxa*. In addition to lignin, these cell walls contain high percentages of cellulose (microfibrillar 1→4‐β‐glucan), low percentages of xyloglucan (XG; 1→4‐β‐glucan substituted by xylosyl residues), mixed‐linkage 1→3, 1→4‐β‐glucan (MLG), and a high abundance of 1→4‐β‐xylan. Xylans frequently contain acetyl, arabinosyl and/or glucuronyl substituents attached to some backbone xylose residues (Carpita, 1996; Scheller & Ulvskov, 2010), hence the designations, arabinoxylan (AX) and glucuronoarabinoxylan (GAX). Grass cell walls also contain small amounts of pectins, which are α‐galacturonate‐rich polysaccharides, and are thought to consist essentially of three interconnected domains joined together by glycosidic bonds: homogalacturonan (HG), rhamnogalacturonan‐I (RG‐I) and rhamnogalacturonan‐II (RG‐II) (O'Neill *et al*., 1990; Fry, 2010; Atmodjo *et al*., 2013). HG makes up most of cell wall pectin and comprises unbranched chains of α‐galacturonate residues, joined by 1→4‐bonds, which may be methyl‐esterified (Atmodjo *et al*., 2013). Additionally, other less abundant components may be found, such as structural proteins and hydroxycinnamates (HCAs). The complexity of cell wall composition is increased by the fact that cell wall components are interconnected via processes that are not yet completely understood (McCann & Carpita, 2015; Park & Cosgrove, 2015), to form molecular structures that maintain the integrity of plant tissues and resist exogenous attack (Pauly & Keegstra, 2008; Chowdhury *et al*., 2014). Consequently, understanding these complex and intricate networks is a daunting task, especially if we consider that *c*. 10% of plant genomes is associated with cell wall assembly and disassembly (Carpita & McCann, 2015).

Research aimed at improving lignocellulosic biomass for biorefining applications has motivated considerable advances in our understanding of plant cell walls. Identification of desirable cell wall characteristics, and the development of crops containing such features, are crucial steps for lignocellulosic biorefining optimization. However, as all cell walls are not identically composed, it is often difficult to systematically assess the quality of a given feedstock in relation to others, particularly between different studies. Here we present a reference cell wall profile for miscanthus leaf and stem biomass harvested at three developmentally distinct time points. This reference profile is particularly focused on cell wall glycan components, given that glycans compose the bulk of grass cell walls and possess the most wide‐ranging potential for industrial valorization (Lucia, 2008; Uraki & Koda, 2015).

A developmentally focused study of structural glycan composition and distribution in miscanthus organs and/or tissues is relevant not only for the optimization of lignocellulosic biomass utilization as a feedstock for renewable bioproduct and bioenergy solutions, but also to improve our fundamental understanding of the cell wall. Previous work based on Fourier transform mid‐infrared spectroscopy (FTIR) studies suggested that structural polysaccharides are major contributors to compositional variability during stem development and between organs (da Costa *et al*., 2014). In the present study, we identify components responsible for this compositional variability and discuss several relevant observations related to composition, structure and possible interactions between cell wall components. Moreover, a large and diverse set of plant glycan‐directed monoclonal antibodies (mAbs) was used to monitor changes in matrix glycan distribution, structure and extractability. This suite of molecular probes possesses enough variability to allow the identification of most classes of noncellulosic plant cell wall polysaccharides (Pattathil *et al*., 2010, 2015). For the first time, this array of glycan‐directed mAbs was used to comprehensively characterize variations in the cell wall glycome of miscanthus across different harvests, organs and genotypes, to generate a representative reference profile for miscanthus cell wall biomass.

Several global breeding programmes focus on harnessing the genotypic and phenotypic variation among and within miscanthus species with the aim of genetically improving miscanthus traits relevant to the enhancement of biomass quality and yield (Heaton *et al*., 2004; Yan *et al*., 2012; Robson *et al*., 2013; van der Weijde *et al*., 2016). The detailed portrait of miscanthus cell wall presented here will contribute to these efforts, as it provides relevant information for the tailoring of miscanthus varieties with preferable characteristics for conversion to biofuels and other biomaterials.

## Materials and Methods

### Plant material

A general characterization of miscanthus cell wall has been reported (da Costa *et al*., 2014). Here we present more detailed studies based on a subset of the genotypes used in the original study: *M*. × *giganteus* (gig01), *M*. *sinensis* (sin08, sin09, sin11, sin13, sin15), *M*. *sacchariflorus* (sac01) and a nonspecified interspecific hybrid (hyb03). Experimental plots and growth conditions have been described previously (Allison *et al*., 2011; da Costa *et al*., 2014).

### Cell wall material

All compositional analyses and *Clostridium phytofermentans* bioassays were performed on purified cell wall material (CWM). Individual leaf and stem samples were collected from single tillers harvested at time points corresponding to three developmental stages: 10 wk after shoot emergence, when plants were actively growing; 18 wk, when plant growth had reduced to a minimal rate, peak biomass; 42 wk, senesced stage. CWM was prepared as described in da Costa *et al*. (2015).

### Neutral monosaccharides

Acid hydrolysis was performed as described in Sluiter *et al*. (2012): 10 mg of CWM were weighed into 10 ml Pyrex glass tubes and 100 μl H_2_SO_4_ (72% w/w) was added. Sealed tubes were left at 30°C for 1 h. Samples were diluted to 4% H_2_SO_4_ (w/w) and autoclaved at 121°C for 1 h. Once at room temperature, tubes were centrifuged to produce a supernatant; 400 μl of diluted (1 : 200) samples was transferred to filter vials (0.45 μm nylon filter; Thomson Instrument Company, Oceanside, CA, USA). Carbohydrate separation was by high‐performance anion‐exchange chromatography with pulsed amperometric detection (HPAEC‐PAD) using a gold working electrode and Ag/AgCl reference electrode. The ICS‐5000 ion chromatography system (Dionex, Sunnyvale, CA, USA) was operated at 45°C using a CarboPac SA10 column with a CarboPac SA10G guard column. An eluent generator prepared 0.001 M KOH for 14 min isocratic elution at 1.5 ml min^−1^. Calibration standards were used for monosaccharide identification and quantitation. In order to resolve galactose (Gal) which coelutes with rhamnose at 45°C, the HPAEC‐PAD method was adapted by reducing the flow rate to 1.2 ml min^−1^ and temperature to 30°C.

### Acetyl esters

Cell wall‐bound acetate was released by an alkaline saponification procedure modified from Manabe *et al*. (2011). CWM (10 mg) was incubated in 500 μl 0.1 M KOH for 16 h with constant shaking (21°C/150 rpm). Samples were centrifuged at 2500 ***g*** for 5 min, 100 μl of the supernatants were mixed with 900 μl 0.005 M H_2_SO_4_ containing 0.005 M crotonic acid as an internal standard. The mixtures were filtered through 0.45 μm syringe filters (Millipore Corporation, Billerica, MA, USA) and 25 μl analysed on a high‐performance liquid chromatography (HPLC) system fitted with a refractive‐index detector (Jasco, Great Dunmow, Essex, UK), equipped with a Rezex ROA‐organic acid H^+^ column (Phenomenex, Torrance, CA, USA), kept at 35°C, with a 0.005 M H_2_SO_4_ mobile phase flowing at 0.6 ml min^−1^ for 16 min. Supernatant acetate concentrations were determined using a concentration gradient of an acetic acid standard.

### Hydroxycinnamoyl esters

Ester‐linked HCAs were released using an alkaline saponification method adapted from Buanafina *et al*. (2006). CWM (10 mg) was mixed with 5 ml 1 M KOH under a flow of N_2_ to reduce sample oxidation, followed by dark incubation for 16 h with constant shaking (21°C/150 rpm). Samples were centrifuged at 2500 ***g*** for 5 min, extracts were transferred to new tubes and pellets were washed with 4 ml methanol (100%). Wash supernatants were combined with the extracts and solubilized carbohydrate was precipitated at −80°C/20 min. After centrifugation (2500 ***g***; 5 min) supernatants were collected in new tubes, pellets were washed with 1 ml methanol (100%) and all supernatants were combined. After methanol evaporation and sample acidification, HCAs were recovered by reverse‐phase C_18_ solid‐phase extraction (Sep‐Pak C_18_ Vac RC cartridges, 500 mg, 3 cm^3^, 55–105 μm; Waters Corporation, Milford, MA, USA). The resulting samples were dried under N_2_ and reconstituted in 200 μl methanol (70% v/v); 20 μl was injected for analysis on a reverse‐phase HPLC system with a diode array detector (RP‐HPLC‐DAD; Waters Corp.) set to collect UV/visible spectra at 240–400 nm. A radial compression column was used (Nova‐Pak C_18_ Radial‐Pak Cartridge, 4 μm particle size; Waters Corp.), with methanol (100%) and acetic acid (5% v/v) as eluents in a 25 min linear 20–70% methanol gradient elution at 2 ml min^−1^. Identity and concentration of monomeric ferulic acid and *p*‐coumaric acid were determined using authentic standards.

### Statistical analysis

All descriptive statistics, ANOVA, Tukey's tests and correlations were calculated using Statistica (v. 8.0; StatSoft, Tulsa, Oklahoma) at a 5% significance level (*α* = 0.05). Effect sizes were calculated as *η* ^2^ statistics (Cohen, 1973; Levine & Hullett, 2002): *η* ^2^ = SS_effect_/SS_total_ (where SS is the sum of squares).

### Glycome profiling

Sequential extractions of CWM, phenol‐sulphuric acid total carbohydrate estimation assays and enzyme‐linked immunosorbent assays of resultant extracts, all part of the glycome profiling study, were carried out as described previously (DeMartini *et al*., 2011; Pattathil *et al*., 2012). Sequential extractions consisted of using increasingly harsher chemicals : ammonium oxalate (0.05 M), sodium carbonate (0.05 M), 1 M KOH, 4 M KOH, acidic sodium chlorite and 4 M KOH PC (postchlorite treatment). The sequential extracts were screened with a comprehensive suite of noncellulosic glycan epitope‐directed mAbs (Pattathil *et al*., 2010) obtained from the Complex Carbohydrate Research Center laboratory stocks (CCRC, JIM and MAC series) or from BioSupplies (Bundoora, Vic., Australia) (BG1, LAMP). Supporting Information Table S1 describes all mAbs used. Heat maps representing glycome profiling data were produced as previously described (Pattathil *et al*., 2012). Principal components analysis (PCA) of glycome profiling data was performed using the Eigenvector PLS Toolbox (v. 7.0.3; Eigenvector Research, Wenatchee, WA, USA) within MatLab (v. R2010b; MathWorks, Natick, MA, USA).

### Lignin measurement

Lignin content of the residues resulting from the sequential extraction was determined using the acetyl bromide procedure, as previously described (da Costa *et al*., 2014).

### *C*. *phytofermentans* bioassay of biomass digestibility

A previously described bioassay (Lee *et al*., 2012a,b) which uses *Clostridium phytofermentans*‐mediated ethanol fermentation yields as digestibility indicators was used to estimate CWM amenability to deconstruction.

### *In situ* immunolabelling

Sections from the middle portions of leaf blades and stem internodes, both located halfway through the length of the tiller, from gig01 (*M*. × *giganteus*) plants were sampled at peak biomass and immersed in a fixative solution consisting of paraformaldehyde (1.6% v/v) with glutaraldehyde (0.2% v/v) in 0.025M sodium phosphate buffer (pH = 7.1).

*In situ* cell wall glycan epitopes were detected by fluorescence immunolabelling according to procedures described elsewhere (Avci *et al*., 2012). Briefly, samples were dehydrated using a graded ethanol series, infiltrated and embedded in LR White resin (Ted Pella, Redding, CA, USA) in gelatine capsules. Resin was polymerized at 4°C under UV light (365 nm). Transverse semithin sections (250 nm) were cut with a Leica EM UC6 ultramicrotome (Leica Microsystems, Buffalo Grove, IL, USA). Sections were blocked with 3% (w/v) nonfat dry milk in 0.01 M potassium phosphate‐buffered saline containing 0.5 M NaCl (KPBS; pH = 7.1) for 30 min and washed with the same buffer. Undiluted hybridoma supernatants containing the mAbs were applied to the sections, incubated for 90 min and then washed with KPBS. Goat anti‐mouse immunoglobulin‐G (IgG) or anti‐rat IgG conjugated to Alexa‐fluor 488 (Invitrogen, Waltham, MA, USA) diluted 1 : 100 in KPBS was applied. After 90 min, sections were washed with KPBS and deionized H_2_O. For certain mAbs known to only bind to de‐esterified forms of the epitopes, sections were subjected to a base treatment with 0.1 M KOH (1 h followed by three washes with deionized H_2_O), before blocking and mAb application. Microscopic inspection was performed using an Eclipse 80i microscope (Nikon, Melville, NY, USA) equipped with epifluorescence optics. Images were captured with a Nikon DS‐Ri1 camera head using NIS‐Elements Basic Research software (Nikon).

## Results

### Relative abundances of cell wall monosaccharides vary significantly in stem and leaf biomass throughout plant growth

Plant cell wall glycans and their monosaccharide constituents represent valuable biorefinery feedstocks. Monosaccharide compositions were determined for each leaf and stem sample from the eight genotypes examined in this study, harvested at three developmental stages: active growth, peak biomass and senescence (Table 1). Further information concerning the distribution of monosaccharide content measurements across eight genotypes is provided in Figs S1 and S2.

**Table 1:** Composition of miscanthus biomass expressed as percentage of cell wall material (CWM) DW (%CWM)

	Active growth		Peak biomass		Senescence	
	Leaf	Stem	Leaf	Stem	Leaf	Stem
Total sugar	61.7 ± 9.9	66.5 ± 9.2	57.3 ± 5.3	63.2 ± 6.3	60.6 ± 5.4	62.2 ± 7.3
Glucose	43.6 ± 6.6	48.4 ± 7.2	40.4 ± 3.8	46.1 ± 4.5	41.8 ± 3.2	45.0 ± 5.3
Xylose	14.2 ± 2.8	16.2 ± 2.5	13.3 ± 1.7	15.2 ± 2.4	15.4 ± 2.2	15.4 ± 2.2
Arabinose	2.89 ± 0.63	1.43 ± 0.32	2.55 ± 0.51	1.36 ± 0.33	2.44 ± 0.35	1.32 ± 0.20
Galactose	0.84 ± 0.18	0.29 ± 0.12	0.88 ± 0.20	0.33 ± 0.11	0.77 ± 0.17	0.34 ± 0.11
Fucose	0.20 ± 0.05	0.22 ± 0.06	0.23 ± 0.05	0.21 ± 0.06	0.22 ± 0.07	0.20 ± 0.07
Ara/Xyl	0.21 ± 0.04	0.09 ± 0.02	0.19 ± 0.04	0.09 ± 0.02	0.16 ± 0.03	0.09 ± 0.02
Lignin	18.1 ± 0.9	19.1 ± 1.8	19.5 ± 0.6	21.8 ± 1.6	22.1 ± 1.5	23.8 ± 0.7
Acetate	3.15 ± 0.32	4.77 ± 0.36	3.36 ± 0.37	4.70 ± 0.38	4.19 ± 0.71	4.66 ± 0.53
*p*‐Coumaric acid	0.75 ± 0.29	1.18 ± 0.63	0.66 ± 0.21	1.14 ± 0.64	0.72 ± 0.25	1.10 ± 0.45
Ferulic acid	0.43 ± 0.17	0.41 ± 0.15	0.32 ± 0.11	0.35 ± 0.14	0.30 ± 0.13	0.32 ± 0.12
Released ethanol	51.8 ± 2.5	49.4 ± 3.9	48.4 ± 2.5	46.2 ± 2.3	39.4 ± 1.4	40.1 ± 1.4

Values are means ± SD of leaf or stem biomass from eight genotypes at each individual developmental stage. Total sugar values represent the mean of the sum of all quantified monosaccharides in each CWM sample collected from leaf or stem at each developmental stage. Ara/Xyl is the mean ratio of arabinose : xylose in the samples. Lignin values were taken from da Costa *et al*. (2014) and means recalculated for the eight genotypes used in the present study, instead of the original 25. Released ethanol values are expressed as mg ethanol g^–1^ dry CWM and consist of supernatant ethanol concentrations after 72 h incubation with *Clostridium phytofermentans*.

Fucose (Fuc) and Gal together represented < 2% of miscanthus CWM dry weight (DW). Glucose (Glc) was the most abundant monosaccharide in miscanthus CWM, followed by xylose (Xyl), comprising, on average, 44.2% and 14.9% DW of all samples analysed, respectively (Table 1). Overall, Glc and Xyl contents were significantly higher in stems than in leaves (*P *<* *0.001). However, Xyl content did not differ significantly (*P *=* *0.880) between senesced leaves and stems (Table 1). Glc abundance differed significantly (*P *<* *0.001) between harvests, as values were higher earlier in development than at senescence. Xyl content also varied significantly (*P *<* *0.001) throughout development, although differently for each organ. In common with Glc trends, the abundance of Xyl in stem cell walls was higher at the first harvest than at senescence. However, in leaves, Xyl content was higher in senesced material than at active growth.

Mean arabinose (Ara) content was *c*. 2% of CWM DW (Table 1). Ara abundance primarily varied according to organ origin (*P *<* *0.001; *η* ^2^ *= *0.6966), with leaves containing almost twice the Ara content of stems. Additionally, Ara abundance varied significantly (*P *<* *0.001) between harvests, decreasing in consecutive harvests. Although Ara and Xyl may originate from different classes of cell wall polysaccharides, in grasses like miscanthus these monosaccharides should derive primarily from AX (Carpita, 1996). Xylan arabinosylation may be estimated by calculating Ara/Xyl ratios (Rancour *et al*., 2012). Organ origin was the main source of variation in Ara/Xyl ratios (*P *<* *0.001; *η* ^2^ *= *0.6841), as values were higher in leaves than in stems by a factor of 2.33 at active growth, 2.11 at peak biomass and 1.78 at senescence (Table 1). In leaves, Ara/Xyl ratios suggested altered arabinosylation degrees as plants matured, with a decline from active growth to peak biomass (−9.5%) and between peak biomass and senescence (−15.8%). By contrast, in stems the ratios did not vary substantially between harvests (Fig. S3).

### Cell wall acetylation increases in leaf biomass as plants mature, but remains constant in stems

The biological role of cell wall polymer acetylation is poorly understood (Manabe *et al*., 2011, 2013), but may be involved in xylan–cellulose interactions (Busse‐Wicher *et al*., 2014), which influence recalcitrance and, subsequently, biomass conversion. Acetate release was measured by 0.1 M KOH‐mediated saponification; that is, acetyl ester linkage cleavage (Marcus *et al*., 2010; Jönsson *et al*., 2013). Significant (*P < *0.001) differences between organs were observed in released acetate abundance. However, the degree of cell wall acetylation did not vary equally in each organ throughout development. Acetate content of leaves differed significantly between developmental stages (*P *<* *0.001), as mean acetylation degree increased in consecutive harvests. By contrast, in the CWM of stems, measured acetate did not change significantly throughout plant development (*P *=* *0.525), with an average value of 4.7% DW at all developmental stages (Table 1; Fig. S4).

### More FA and *p*CA are observed in younger plant cell walls

Grass cell wall polymers are abundantly feruloylated and *p*‐coumaroylated (Carpita, 1996). Ferulic acid (FA), which is predominantly ester‐linked to AX and GAX, has been associated with digestibility, while *p*‐coumaric acid (*p*CA) appears mostly esterified to lignin (Lu & Ralph, 1999). Ester‐linked hydroxycinnamoyl quantitation was performed after cell wall saponification with 1 M KOH. FA content was not significantly different between leaf and stem (*P *=* *0.153), but at active growth, mean FA content was higher than in later harvests by at least 34% in leaves, and 17% in stems (Table 1; Fig. S5). Conversely, *p*CA release was significantly different between organs (*P *<* *0.001), with mean *p*CA content in stems being 50% higher than in leaves (Table 1).

### A reference cell wall glycome profile allows identification of key glycan modifications in the cell wall of developing plants

Changes in matrix glycan composition, structure and extractability were monitored in miscanthus CWM using glycome profiling, which employs increasingly harsh, sequential multistep extractions to fractionate the cell wall (Pattathil *et al*., 2012; Li *et al*., 2014). Most noncellulosic glycans were extractable before biomass delignification with acidic sodium chlorite, and the 1 M KOH fractions contained the largest portion of extracted glycans. Total released carbohydrate present in each extract and the composition of the residues remaining after cell wall fractionation are shown in Fig. S6 and Tables S2, S3.

**Figure 1:**
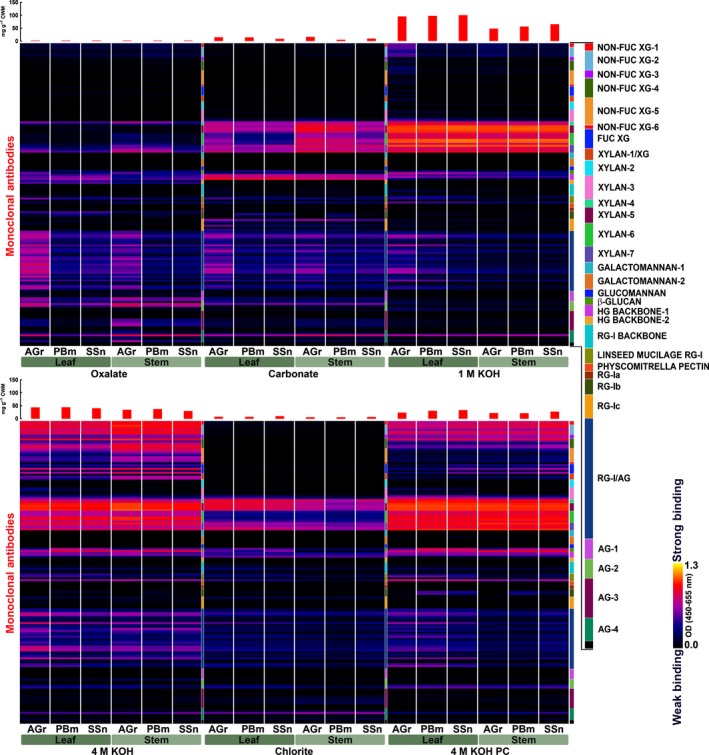
Glycome profile of miscanthus cell wall material (CWM) extracted sequentially with ammonium oxalate, sodium carbonate, 1 M KOH, 4 M KOH, sodium chlorite and 4 M KOH postchlorite treatment (PC) as explained in the Materials and Methods section. Corresponding organs and developmental stages are labelled below each profile: AGr, actively growing; PBm, peak biomass; SSn, senesced. Each extract was probed against an array of plant glycan‐directed monoclonal antibodies (Supporting Information Table S1 for a list of all mAbs). Antibody binding strength based on optical density (OD) is presented as a colour gradient ranging from black (no binding) and red (intermediate binding), to yellow (strongest binding) (OD = 1.3). Binding intensities are the averages of all genotypes studied here. Red bars on the top indicate the average amount of sugars recovered in the solubilized extracts g^–1^ CWM (mg g^−1^).

Glycome profiling data are summarized as heat maps, where the mean (Fig. 1), and the standard deviation (Fig. S7) of binding intensities for each glycan‐directed mAb across all miscanthus genotypes are depicted for each harvest and organ.

Antibodies directed at XG epitopes showed highest binding signals in the 4 M KOH extracts (both pre‐ and post‐sodium chlorite treatment) with more diverse and higher abundance of XG epitopes being removed from stems than from leaves, particularly before delignification. This is exemplified by the significantly increased abundance of XG epitopes recognized by nonfucosylated (nonfuc) XG‐4, nonfuc XG‐5 and xylan‐1/XG groups of mAbs. Among the XG epitopes extracted with 1 M KOH, those detected by the nonfuc XG‐1 and nonfuc XG‐2 groups of mAbs were more abundant in active growth leaf samples than in mature leaves, or in stems.

Xylan epitopes were found in all extracts, but their presence and abundance varied in different organs and harvests. For the most weakly integrated xylans present in oxalate extracts, epitopes bound by the xylan‐4 and xylan‐7 mAb subclasses were present in leaf and stem, but marginally more abundant in actively growing stems. High abundance of xylan epitopes was apparent in most carbonate extracts, with the exception of leaves from peak biomass and senesced harvests. In these cases, there were lower signals for xylan‐6 mAbs. In the case of more tightly bound xylans, released with 1 M and 4 M KOH (both pre‐ and postchlorite), epitopes recognized by the xylan‐4 through xylan‐7 groups of mAbs were highly abundant. In general, no substantial variations in the abundance of these epitopes were noted between organs or developmental stages. Interestingly in chlorite extracts, a reduced abundance of xylan epitopes that are specifically recognized by the xylan‐6 groups of mAbs was detected.

Binding intensities of mAbs directed at 1→3‐β‐glucan (callose) and MLG increased with harsher extractions and were more abundant in leaves. Exceptions are the 4 M KOH extracts, where binding was similar between organs.

Homogalacturonan backbone‐1 epitopes occurred in all fractions with few developmental and organ differences, except for slightly higher binding intensities in the oxalate and 1 M KOH extracts from leaves. RG‐I backbone epitope detection was low, almost exclusively perceptible in strong alkaline and chlorite extracts, but subtly more abundant in leaf extracts. The RG‐I/AG class of mAbs, which detects pectic arabinogalactan epitopes, in general showed higher abundance in oxalate, carbonate and 1 M KOH extracts from active growth and, overall, leaf extracts exhibited marginally increased abundance of these epitopes compared with stem samples. A higher abundance of these epitopes in leaves from all developmental stages is also exhibited in 4 M KOH PC extracts. Glycan epitopes bound by the AG‐1 to AG‐4 groups of mAbs may occur in arabinogalactan‐containing polysaccharides as well as in arabinogalactan proteins (AGPs). For these mAbs, the highest signals were detected in oxalate extracts from actively growing plants, mainly in stems. In chlorite extracts, especially from stems, binding signals for some AG‐4 mAbs were increased, suggesting that a proportion of these epitopes could not be released before delignification.

### *In situ* immunolabelling revealed organ‐specific distribution patterns of cell wall glycan epitopes

Immunohistochemistry of *M*. × *giganteus* stem and leaf midrib sections using key mAbs recognizing distinct glycan epitopes (Table 2) revealed epitope distributions at the cellular level, thus verifying glycome profiling results *in situ*.

**Table 2:** Cell wall glycan‐directed monoclonal antibodies (mAbs) used in the study of *in situ* immunolabelling of *Miscanthus* × *giganteus* leaf and stem tissues (further information on all mAbs used here can be found in Supporting Information Table S1)

mAb	mAb subclass – based on Pattathil *et al*. (2010)
CCRC‐M88	Nonfucosylated xyloglucan‐2
CCRC‐M1	Fucosylated xyloglucan
CCRC‐M154	Xylan‐4 (arabinosylated xylan)
CCRC‐M155^BT^	Xylan‐5 (Me‐GlcA substituted xylan)
CCRC‐M149	Xylan‐7 (xylan backbone DP≥4)
BG1	Mixed‐linkage (1→3, 1→4)‐β‐glucan
CCRC‐M38	Demethyl‐esterified homogalacturonan backbone‐1
CCRC‐M128	RG‐I/AG (arabinogalactan side chains of rhamnogalacturonan‐I)

^BT^Used in combination with a 0.1 M KOH base treatment of the sections before immunolabelling.

**Figure 2:**
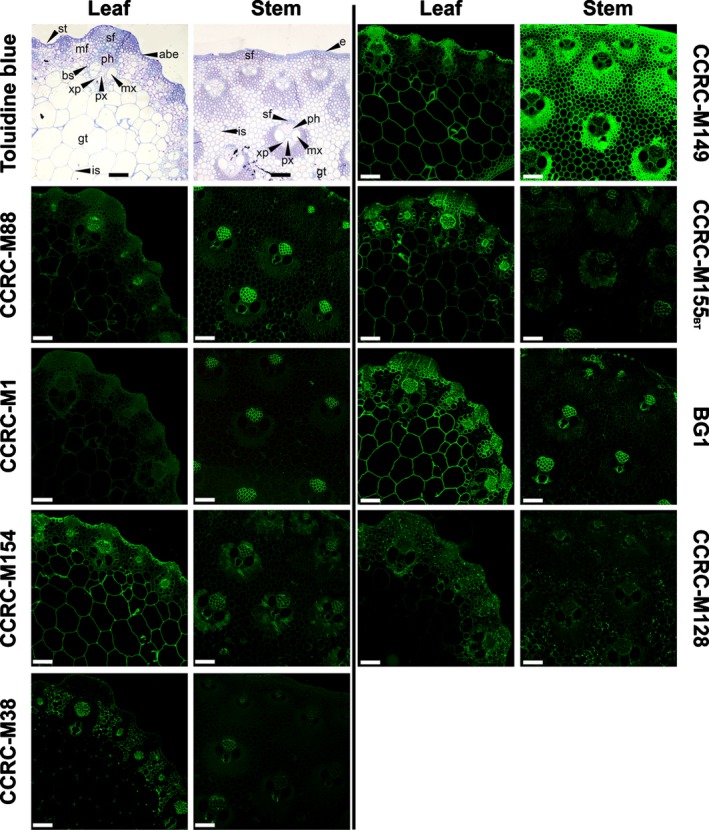
Immunofluorescent labelling of cell wall glycan epitopes in transverse sections from leaves and stems from *Micanthus* × *giganteus* (gig01). Immunolabelling studies were preceded by microscopic inspection of sections stained with toluidine blue to characterize their histological complexity. Used mAbs: CCRC‐M88, nonfucosylated xyloglucan (XG); CCRC‐M1, fucosylated XG; CCRC‐M154, Ara‐substituted xylan (Xylan‐4); CCRC‐M149, xylan backbone (Xylan‐7); CCRC‐M155, Me‐GlcA‐substituted xylan (Xylan‐5 de‐esterified epitopes); BG1, mixed‐linkage (1→3, 1→4)‐β‐glucan; CCRC‐M128, arabinogalactan side chains of rhamnogalacturonan‐I (RG‐I/AG); CCRC‐M38, demethyl‐esterified homogalacturonan (HG‐backbone‐1). More details regarding the mAbs are available in Supporting Information Table S1. For CCRC‐M155, results are shown for immunolabelling of sections after a base treatment (BT) with 0.1 M KOH, which removed ester‐linked substituents from the walls (nonbase‐treated control for CCRC‐M155 may be found in Fig. S8). abe, abaxial surface epidermis; bs, bundle sheath; e, epidermis; gt, parenchymatous ground tissue; is, intercellular space; mf, mesophyll cells; mx, metaxylem; px, protoxylem; sf, sclerenchyma fibres; st, stomatal complex; xp, xylem parenchyma. Bars, 100 μm.

Nonfucosylated XG epitopes probed with CCRC‐M88 revealed strong labelling in phloem but fainter labelling in xylem of leaves and stems (Fig. 2). However, fluorescence patterns differed between organs, as labelling of parenchymatous and sclerenchymatous structures was more perceptible in stems. Probing of fucosylated XG with CCRC‐M1 showed more abundant labelling in stem cell walls, but mostly restricted to phloem.

Xylan‐directed mAbs CCRC‐M154 (xylan‐4), CCRC‐M149 (xylan‐7) and CCRC‐M155 (xylan‐5) displayed varying labelling patterns in different organs (Fig. 2). With CCRC‐M154, which is selective for arabinose‐substituted xylans (Schmidt *et al*., 2015), labelling was low in stems, but was detected in most leaf cell walls, although more intensely in the phloem. CCRC‐M149, which binds to the xylan backbone, abundantly labelled cell walls of most cell types, but less in leaf mesophyll and interfascicular parenchyma. Labelling of epidermal and sclerenchymatous thickenings was particularly visible. Very low reactivity was detected initially with CCRC‐M155, which binds to a 4‐O‐Me GlcA‐substituted xylan epitope (M. G. Hahn *et al*., unpublished) (Fig. S8). All of the xylan‐directed mAbs used in this study were generated using alkali‐extracted de‐esterified xylans and therefore may not recognize acetylated xylan epitopes *in vivo* (Li *et al*., 2014). To test for this, 0.1 M KOH was employed to remove ester‐linked groups in the organ sections, thus rendering xylan epitopes accessible. Epitope recognition by CCRC‐M155 became abundant after this base treatment (Fig. 2), revealing where at least some esterified xylan epitopes occur in intact cell walls. Labelling was predominant in phloem and xylem parenchyma, although more widespread patterns were apparent in leaves, including walls of bundle sheath, epidermal and stomatal subsidiary cells. Additionally, labelling was more intense in portions of the outer layers of the walls in foliar parenchymatous ground tissue.

In accordance with glycome profiling results, labelling with CCRC‐M174 (galactomannan‐directed) was negligible in analysed sections (Fig. S8).

The BG1 mAb, which binds MLG epitopes (Meikle *et al*., 1994), showed more intense labelling patterns in leaf sections (Fig. 2), where fluorescence was visible in most cell types, except in walls of metaxylem, mesophyll and stomatal subsidiary cells.

Labelling of AG side chains of RG‐I with CCRC‐M128 was concentrated in membranous structures. De‐methyl‐esterified HG epitope distributions probed with CCRC‐M38 were similar in the vascular bundles of stems and leaves, as only phloem and protoxylem were labelled (Fig. 2). However, patterns differed between organs, as leaf parenchyma and mesophyll showed higher accumulation of CCRC‐M38 epitopes, particularly on middle lamellae and lining intercellular spaces of cell junctions.

### Miscanthus biomass amenability to conversion depends on organ and harvest

A *C. phytofermentans* bioassay was employed to estimate how miscanthus CWM recalcitrance varied between leaf and stem samples and throughout plant maturation. Mean released ethanol yields, expressed as mg ethanol g^–1^ cell wall DW, ranged from 40.07 mg g^−1^ in senesced stem to 51.83 mg g^−1^ in active growth leaf. Ethanol yield decreased as plants matured and was higher in leaves than in stems, except for senesced samples, where the values did not vary substantially (Table 1).

## Discussion

### Miscanthus cell wall glycans

**Figure 3:**
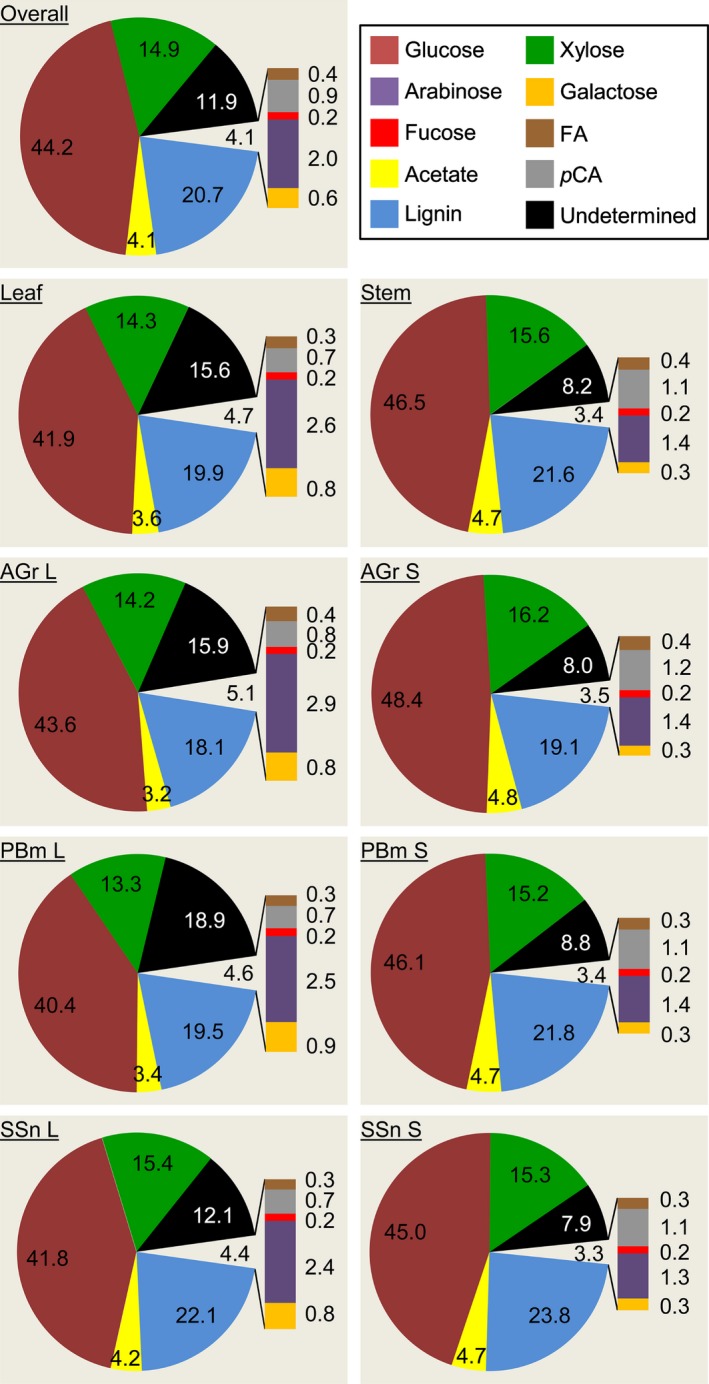
Mean percentage (%) composition of miscanthus cell wall biomass DW. Overall indicates the composition for leaf and stem at all developmental stages combined. Lignin values are reproduced from da Costa *et al*. (2014). Undetermined components may include uronic acids, ferulate dimers and oligomers, methyl groups, proteins and minerals. FA, ester‐linked ferulic acid; *p*CA, ester‐linked *p*‐coumaric acid; AGr, active growth; PBm, peak biomass; SSn, senescence; L, leaf; S, stem.

Structural polysaccharides are major contributors to compositional differentiation between miscanthus organs and harvests (da Costa *et al*., 2014). Here, we explored these differences further and identified typical cell wall characteristics of miscanthus organs from different harvests (summarized in Fig. 3).

In Poales cell walls, Glc is primarily derived from cellulose, but some Glc is also found in MLG and XG, which are in much lower abundance in Poales walls (Carpita, 1996). The higher Glc content in stems than in leaves, and in younger plants than at senescence, shown in this study have also been reported elsewhere for miscanthus (Le Ngoc Huyen *et al*., 2010; van der Weijde *et al*., 2016) and for *Brachypodium distachyon* (Rancour *et al*., 2012). Given the supportive role of stems, higher total sugar content implies more abundant structural polysaccharides, which make up the major load‐bearing network in plant cell walls (Harris & Stone, 2008; Xu, 2010; Leroux, 2012).

The combined Ara and Xyl contents suggest that the abundance of AX, which is the principal glycan from the Poales walls from which these monosaccharides arise, does not vary considerably between organs. However, Ara content and Ara/Xyl ratios were approximately twofold higher in leaves, suggesting higher arabinosylation than in stems (Table 1; Fig. S3). This agrees with higher AX branching reported in *Saccharum officinarum* leaves than in culms (de Souza *et al*., 2013). It has also been shown that highly substituted AX is prominent in primary cell walls, while less substituted xylans are associated with secondary cell wall and lignification (Suzuki *et al*., 2000).

In grasses, pectins and AGPs are the main Gal‐containing cell wall polysaccharides (Carpita, 1996; Ishii, 1997; O'Neill & York, 2003). Higher Gal in foliar biomass may thus suggest more abundant pectin and/or AGPs in leaves than in stems.

### Key glycome modifications in miscanthus cell walls

**Figure 4:**
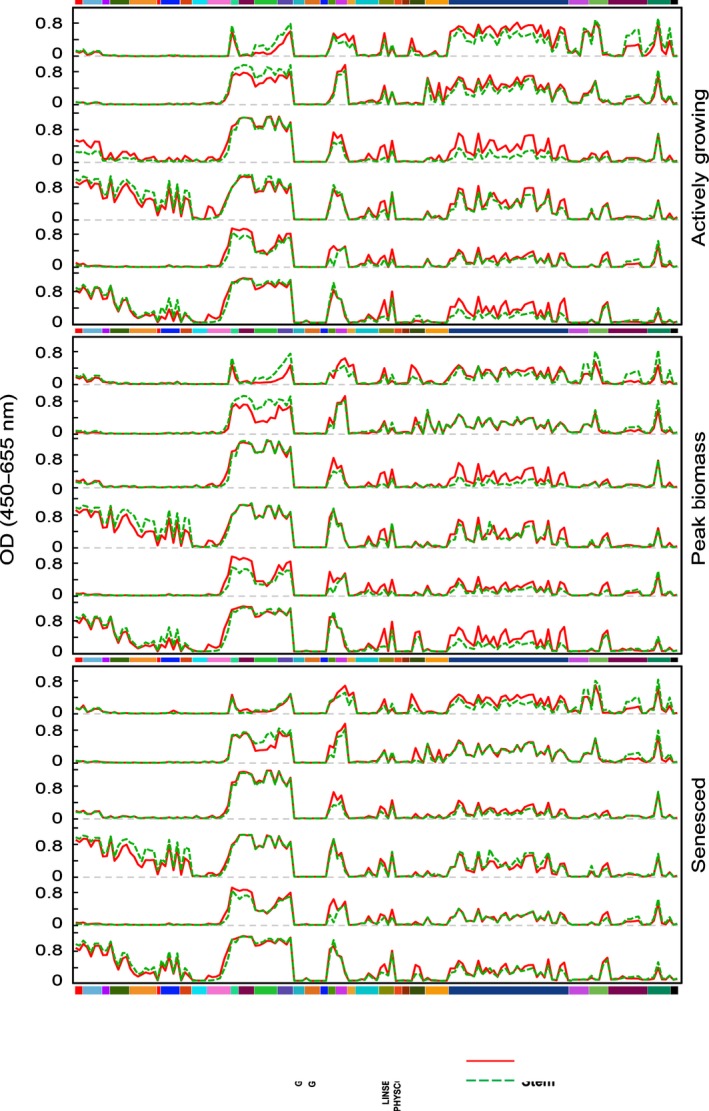
Mean binding values to different classes of cell wall glycan epitopes released at sequential extraction steps from leaf and stem samples from miscanthus biomass at three developmental stages (cf. Fig. 1 and Supporting Information Fig. S9).

A miscanthus reference glycome profile (Fig. 1) represents a powerful tool for future studies, allowing comparisons with other genotypes or lignocellulosic feedstocks. To facilitate comparisons between extracts, organs and harvests, this reference profile was graphically reinterpreted (Figs 4, S9). Furthermore, the most heterogeneous portions of the cell wall glycome are highlighted in standard deviation heat maps (Fig. S7). To clarify the significance of these heterogeneities and determine glycan classes that contribute to the distinctiveness between organs and harvests, PCA was performed as a discriminant tool.

#### Increasingly harsher extractants produce qualitatively distinct cell wall fractions, demonstrating that different polysaccharide classes are not equally integrated into the overall cell wall structure

**Figure 5:**
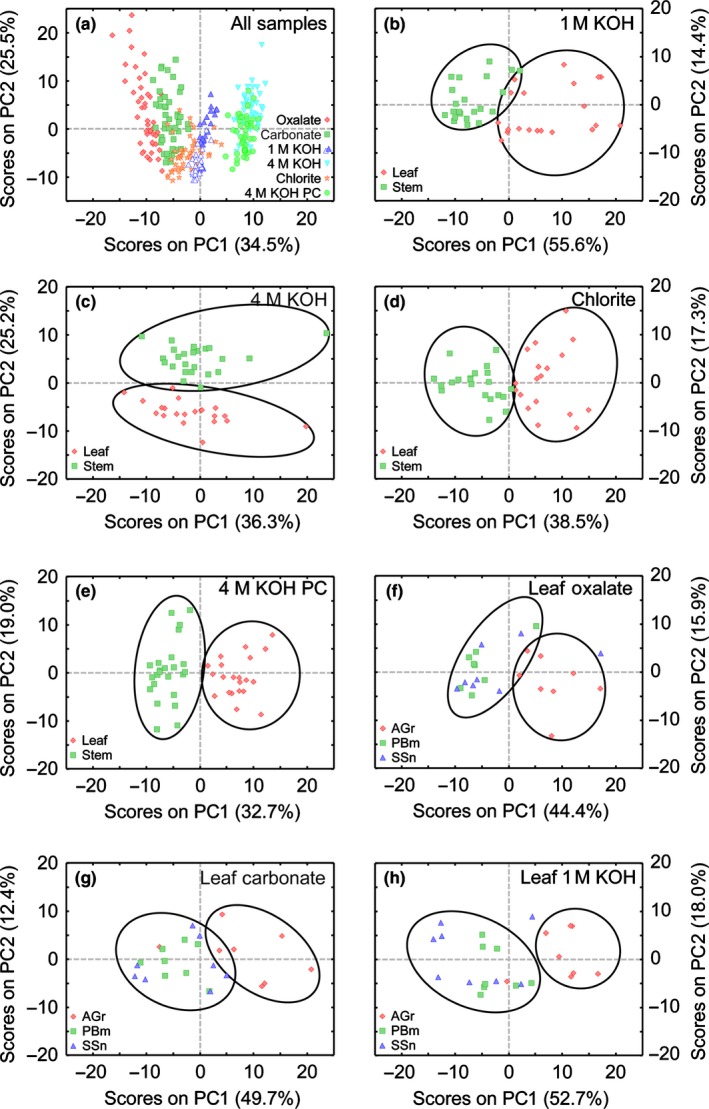
Principal components analysis of glycome profiling data. Plot of principal component 1 (PC1) and principal component 2 (PC2) scores for all samples (a; open triangles in the 1 M KOH extract are stem samples, and blue‐filled triangles are leaf samples), for samples extracted with 1 M KOH (b), 4 M KOH (c), sodium chlorite (d) and 4 M KOH postchlorite (PC) treatment (e); (f, g, h) leaf samples extracted with ammonium oxalate (f), sodium carbonate (g) and 1 M KOH (h). Ellipses are intended to highlight the structure of the data points. AGr, active growth; PBm, peak biomass; SSn, senesced stage.

The various cell wall extracts formed clusters along principal component one (PC1; Fig. 5a). PC1 loadings (Fig. S10) indicated that clusters on the negative side of PC1 (oxalate and carbonate) correspond to extracts with typically stronger binding by pectic epitope‐directed mAbs. Conversely, clusters on the positive side of PC1 (4 M KOH and 4 M KOH PC) showed higher binding by XG‐ and xylan‐directed mAbs. These results suggest that there are differences between the walls in leaf vs stem cells in terms of how firmly different polysaccharide types are integrated into the wall matrix. In chlorite extracts, xylan‐directed mAbs showed higher signals, but MLG and various pectin epitopes were also detected. MLG binding signals were detected in all 1 M KOH extracts, but were more abundant in actively growing plants. This agrees with findings that, despite higher MLG accumulation in younger plants, it is also found in mature organs (Vega‐Sánchez *et al*., 2013). As MLG is released using extractants of different stringency, varying solubility may depend on ratios between trisaccharide/tetrasaccharide MLG domains (Collins *et al*., 2010; Vega‐Sánchez *et al*., 2015).

#### Miscanthus leaves and stems present distinct matrix cell wall glycomes and hence have different overall wall structures

For each sequential extraction step, a PCA model revealed two separate clusters: one comprising glycome profiles from stems, and another from leaves (Figs 5b–e, S11). Depending on the PC responsible for the separation, corresponding loadings identified principal glycan contributors to organ‐dependent variation. For leaf oxalate and carbonate extracts, higher RG‐I/AG epitope binding is a main diverging factor in relation to stems. In carbonate extracts, binding to easily extracted xylan epitopes is also a discriminant feature between organs, as stem samples exhibit stronger binding by xylan‐5, xylan‐6 and xylan‐7 groups of mAbs. In 1 M KOH extracts, organ differentiation primarily derives from higher signals in leaves for most glycan epitopes, particularly for pectins and β‐glucans. For 4 M KOH extracts (pre‐ and postchlorite), stems typically showed higher binding for XG and xylan‐5, xylan‐6 and xylan‐7 mAbs. In leaves, 4 M KOH extracts showed higher binding for most xylan‐3, xylan‐4 and RG‐I mAbs.

#### Stems and leaves are differently modified throughout development

Individual organ models revealed no PCA clusters for any of the stem extracts nor for the three harshest leaf cell wall extracts (Fig. S12), implying that epitope abundance variation for these samples is not significant between harvests. However, in oxalate, carbonate and 1 M KOH leaf extracts, differences in noncellulosic glycan epitope abundances were sufficient to create two clusters, one comprising peak biomass and senesced samples (overlapped), and another from active growth (Fig. 5f–h).

Principal components analysis of glycome profiling data highlighted significant variation between harvests and organs. These differences agree with FTIR‐based predictions that structural polysaccharides are major contributors to developmental and organ‐dependent compositional variation in miscanthus lignocellulosic biomass (da Costa *et al*., 2014). Considering both studies, it appears that cell wall compositional variation between mature and immature plants is primarily associated with nonmatrix cell wall components in stems, namely cellulose and lignin (da Costa *et al*., 2014). In leaves, overall carbohydrate composition varies less than in stems, but significant differences are seen in more easily extractable and less abundant noncellulosic glycan epitopes.

#### Tightly bound glycan epitopes and biomass recalcitrance

Glycome profiling suggests that certain noncellulosic polysaccharide components (namely, subclasses of XGs, heteroxylans, MLG and pectins) are more firmly integrated in the cell wall matrix than others. Knowledge of the structural associations of these populations of glycans with other cell wall constituents is important to improve our understanding of structural features underpinning cell wall recalcitrance.

As sodium chlorite and 4 M KOH PC extracts contain glycans thought to be either directly or indirectly associated with lignin in the cell wall matrix, glycan epitopes detected in these extracts are particularly informative with regard to cell wall recalcitrance. Generally, signals in these extracts did not change notably during development, but binding to most glycan epitopes was higher in chlorite extracts from leaf material than from stems (Figs 4, S11). Most obviously for mAbs in the xylan‐4, xylan‐5, xylan‐7, MLG and several pectin‐directed groups, the glycome profiling data suggest that delignification generally removes more of these glycan epitopes from leaf cell walls than from stem. HG epitopes released with sodium chlorite and 4 M KOH PC (Fig. 4) suggest a pectin–lignin association, corroborated by the higher signals for some RG‐I and RG‐1/AG‐related mAbs in these extracts. Pectin–lignin associations have been suggested for several species: *M. sinensis* (de Souza *et al*., 2015), *Populus trichocarpa* (DeMartini *et al*., 2011), *Panicum virgatum* (Shen *et al*., 2013) and *Medicago sativa* (Wi *et al*., 2005). Abundant MLG epitopes in 4 M KOH PC extracts suggest that all harvests contain proportions of tightly bound MLG, only released after delignification. High MLG detection in harsher extracts was also observed in glycome profiles of switchgrass (Shen *et al*., 2013), sugarcane (de Souza *et al*., 2013) and corn stover (Li *et al*., 2014), and may derive from tight MLG–cellulose associations (Carpita, 1996; Fry, 2010; Kiemle *et al*., 2014). XG epitopes, particularly nonfuc XG‐1, nonfuc XG‐2 and nonfuc XG‐3 groups of mAbs, were prominent in postchlorite extracts, suggesting that despite their reduced abundance in grasses, structural features associated with XG may inhibit sugar release from the cell wall matrix. Considering all extracts, and irrespective of organ origin or developmental stage, signals for the xylan‐4 to xylan‐7 groups of mAbs (which recognize diverse xylan epitopes) were most prominent in postchlorite extracts. Hence, heteroxylans are likely to play important roles in biomass recalcitrance, perhaps as a result of the formation of ferulate–polysaccharide–lignin complexes that crosslink the cell wall (Buanafina, 2009), and/or interactions between arabinoxylan and cellulose.

### Key dissimilarities between organs detected via glycome profiling were verified by immunohistochemistry

*In situ* immunolabelling was performed using mAbs aimed at epitopes from the main noncellulosic miscanthus cell wall glycan classes, and with distinct extractabilities, as revealed by glycome profiling. The resulting labelling patterns suggest that distinct glycan distributions are associated with organ and tissue structural requirements, which may influence biomass recalcitrance to deconstruction.

More broadly disseminated XG epitopes in stem sections compared with leaves may derive from the higher abundance of these glycans in stems, as observed in glycome profiling. As XG is strongly wall‐bound and presumed to interact with cellulose (Hayashi, 1989), XG may be more relevant for stem cohesiveness than for leaves.

Xylan‐directed mAbs showed distinct labelling patterns, suggesting that *M*. × *giganteus* cell wall xylan abundance and structure vary depending on cell type. Labelling of primary walls of *M*. × *giganteus* sections (Fig. 2) with CCRC‐M154, which binds arabinosylated xylans (Schmidt *et al*., 2015), is consistent with reports that GAX occurs in interstitial spaces between primary cell wall cellulose microfibrils (McCann & Carpita, 2008; Li *et al*., 2014). When used with a base treatment, which removes ester‐linked substituents, epitopes recognized by CCRC‐M155 are localized, in particular, to the phloem. By contrast, fewer of the xylan backbone epitopes detected by CCRC‐M149 are present in the phloem, but more are present in other cell walls, particularly where sclerified. Overall, these results suggest that phloem xylans are more substituted than those in sclerenchyma. In *M. lutarioriparius* (Cao *et al*., 2014), sugarcane (de Souza *et al*., 2013) and maize (Suzuki *et al*., 2000), phloem is less lignified than sclerenchyma. Consequently, lignified cell walls may contain less substituted xylans, while more substituted polymers coincide with lower lignification. Indeed, low‐branched xylans occur in all lignified walls, while most highly substituted xylans occur in unlignified tissues (Suzuki *et al*., 2000). As lignin–carbohydrate associations affect cell wall recalcitrance to deconstruction, xylan substitution and localization pattern might influence lignocellulose biorefining.

Reported MLG distribution patterns (Trethewey & Harris, 2002; Vega‐Sanchez *et al*., 2012; Xue *et al*., 2013) are consistent with BG1 labelling of sclerenchyma, phloem and protoxylem cell walls (Fig. 2). To withstand the pressure and tension required for plant support and vascular functions, the cell walls of these organs must be structurally robust. MLG in sclerenchyma, phloem and protoxylem may indeed be contained in the tightly bound MLG epitopes detected by glycome profiling in the KOH and sodium chlorite extracts (Fig. 4), and contribute to structural cohesiveness. Similarly to MLG, leaf sections showed more abundant demethyl‐esterified HG labelling with CCRC‐M38 (Fig. 2). HG demethyl‐esterification promotes the formation of rigid pectin structures, enhancing cell adhesion (Zhang & Staehelin, 1992; Mohnen, 1999; Anthon & Barrett, 2006; Lionetti *et al*., 2010). Furthermore, HG demethyl‐esterification may increase in response to cellulose depletion (Burton *et al*., 2000; Manfield *et al*., 2004; Wolf *et al*., 2009). Given potential cell wall assembly roles, it is pertinent to clarify if recalcitrant MLG and demethyl‐esterified HG in leaves are adaptations for increased cell wall cohesiveness in organs with lower cellulose content.

### Biomass recalcitrance may be affected by several cell wall features beyond lignin content

We have previously reported that the degree of miscanthus biomass lignification does not completely account for biomass digestibility (da Costa *et al*., 2014). In the present study, we expand this conclusion and reveal that several cell wall features may affect recalcitrance, particularly in leaf biomass, as evidenced by correlations with *C*. *phytofermentans* fermentation yields (Table S4).

Acetylation, in particular, shows strong negative relationships with ethanol yields from leaves (*r *=* *−0.52; *P *<* *0.01), suggesting that acetylation is associated with recalcitrance‐enhancing cell wall features. Saccharification and fermentation limitation may originate from acetyl‐mediated steric hindrance to binding of hydrolytic enzymes (Biely, 2012; Pawar *et al*., 2013) and/or microbial inhibition (Gille & Pauly, 2012). Furthermore, acetylation degrees may affect secondary cell wall xylan–cellulose interactions (Busse‐Wicher *et al*., 2014) and influence plant development, as supported by stunted growth phenotypes in *Arabidopsis* mutants with reduced wall acetylation (Manabe *et al*., 2013).

Correlations of ethanol yields with Glc and Xyl content, or Ara/Xyl ratios, highlight the importance of carbohydrate structural features for biomass recalcitrance, supported by glycome profiling results. Strong XG–cellulose associations (Hayashi, 1989) concur with XG epitopes only being extensively removed from the walls when using 4 M KOH. As XG is more abundant in stems, this may contribute to their higher biomass recalcitrance. Moreover, distinct immunolabelling patterns may influence cell wall deconstruction; for example, as cellulose and lignin are lower in leaves, more abundant demethyl‐esterified HG may reinforce organ cohesiveness. Consequently, as pectins are among *C*. *phytofermentans* primary polysaccharide targets (Lee *et al*., 2012b), higher pectin content could make foliar biomass more prone to degradation. Additionally, as HG demethyl‐esterification promotes recalcitrance (Lionetti *et al*., 2010) and methyl‐esterified HG is progressively reduced *in muro* (Zhang & Staehelin, 1992; Mohnen, 1999), more abundant demethyl‐esterified HG in later harvests may contribute to lower ethanol yields.

### Conclusions

Key differences in miscanthus cell wall fine structures and composition were highlighted between organs and harvests. Between harvests, stem cell wall compositional variability is primarily seen in cellulose and lignin, but in leaves it is in easily extractable matrix glycans. Harvest‐ and organ‐specific abundance, distribution, composition and ornamentation of cell wall polymers affect amenability to deconstruction differently. Despite higher stem sugar contents, extracted carbohydrate and ethanol yields were higher from leaves. As a result, the same lignocellulosic feedstock may present widely distinct biorefining potential depending on plant developmental stage and relative organ contributions to total harvested biomass. Understanding how cell wall components interact with each other and altogether respond to biorefining approaches is vital to optimize lignocellulosic biomass deconstruction. Thus, a holistic view of the cell wall is promoted, considering that certain components have variable impacts on recalcitrance depending on overall cell wall assembly. A detailed exploration of factors underpinning recalcitrance will be the future focus of this ongoing study.

## Author contributions

R.M.F.d.C., A.W. and M.B. planned and designed the research. R.M.F.d.C. performed most experiments and all data analyses. Glycome profiling and immunohistochemistry were performed in collaboration with Dr Ronald Clay, U.A. and S.P., under the supervision of M.G.H. S.J.L. performed the *Clostridium* bioassays under the supervision of S.P.H. The manuscript was produced by R.M.F.d.C. and revised by S.P., U.A., S.J.L., S.P.H., A.W., M.G.H. and M.B.

## Supporting information

Please note: Wiley Blackwell are not responsible for the content or functionality of any Supporting Information supplied by the authors. Any queries (other than missing material) should be directed to the *New Phytologist* Central Office.

**Fig. S1** Distribution of measurements of the minor monosaccharides fucose (Fuc) and galactose (Gal) released upon acid hydrolysis of miscanthus CWM.
**Fig. S2** Distribution of measurements of the major monosaccharides arabinose (Ara), glucose (Glc) and xylose (Xyl) released upon acid hydrolysis of miscanthus CWM.
**Fig. S3** Distribution of measurements of the arabinose to xylose ratio (Ara/Xyl) of miscanthus CWM.
**Fig. S4** Distribution of measurements of released acetate upon 0.1 M KOH treatment of miscanthus CWM.
**Fig. S5** Distribution of measurements of ferulic (FA) and *p*‐coumaric (*p*CA) acid released upon 1 M KOH treatment of miscanthus CWM.
**Fig. S6** Total carbohydrate recovered from each sequential extraction step g^–1^ purified cell wall material (mg g^−1^ CWM) estimated by the phenol‐sulphuric acid assay.
**Fig. S7** Heat map of the standard deviations (SD) from the mean binding intensities shown in Fig. 2.
**Fig. S8** Immunofluorescent labelling of cell wall glycan epitopes in transverse sections from leaves and stems from *M. × giganteus* (gig01) with CCRC‐M174 (galactomannan‐2) and before a base treatment with 0.1 M KOH for CCRC‐M155 (xylan‐5, Me‐Glc substituted xylan).
**Fig. S9** Mean binding values to different classes of cell wall glycan epitopes released at sequential extraction steps from leaf and stem samples from miscanthus biomass at three developmental stages (same data as in Fig. 5, but organized by organ).
**Fig. S10** Principal components analysis of glycome profiling data (data are presented for all samples from the six fractions obtained during the sequential extraction).
**Fig. S11** Principal components analysis of glycome profiling data (data is presented for each individual extraction step performed during the sequential extraction).
**Fig. S12** Principal components analysis of glycome profiling (data presented independently for each organ).
**Table S1** Listing of plant cell wall glycan‐directed monoclonal antibodies (mAbs) used in the glycome profiling screening
**Table S2** Amount of carbohydrate recovered at each extraction step g^–1^ of isolated cell wall material (mg g^−1^ CWM) based on phenol‐sulphuric acid assay for total sugar estimation
**Table S3** Monosaccharide and acetyl bromide soluble lignin contents of the residue left after the sequential extraction
**Table S4** Pearson coefficients and associated probability (*P*) of the correlations between the results obtained for the *C. phytofermentans*‐mediated digestibility assessment assay and several cell wall features
