# Supplementary material for: A cell wall reference profile for Miscanthus bioenergy crops highlights compositional and structural variations associated with development and organ origin
Source: New Phytol. 2016 Nov 15;213(4):1710–25. doi: 10.1111/nph.14306 (PMC5324610; doi:10.1111/nph.14306)
Supplement: Supplementary file 1 — Fig. S1 Distribution of measurements of the minor monosaccharides fucose (Fuc) and galactose (Gal) released upon acid hydrolysis of miscanthus CWM. Fig. S2 Distribution of measurements of the major monosaccharides arabinose (Ara), glucose (Glc) and xylose (Xyl) released upon acid hydrolysis of miscanthus CWM. Fig. S3 Distribution of measurements of the arabinose to xylose ratio (Ara/Xyl) of miscanthus CWM. Fig. S4 Distribution of measurements of released acetate upon 0.1 M KOH treatment of miscanthus CWM. Fig. S5 Distribution of measurements of ferulic (FA) and p‐coumaric (pCA) acid released upon 1 M KOH treatment of miscanthus CWM. Fig. S6 Total carbohydrate recovered from each sequential extraction step g–1 purified cell wall material (mg g−1 CWM) estimated by the phenol‐sulphuric acid assay. Fig. S7 Heat map of the standard deviations (SD) from the mean binding intensities shown in Fig. 2. Fig. S8 Immunofluorescent labelling of cell wall glycan epitopes in transverse sections from leaves and stems from M. × giganteus (gig01) with CCRC‐M174 (galactomannan‐2) and before a base treatment with 0.1 M KOH for CCRC‐M155 (xylan‐5, Me‐Glc substituted xylan). Fig. S9 Mean binding values to different classes of cell wall glycan epitopes released at sequential extraction steps from leaf and stem samples from miscanthus biomass at three developmental stages (same data as in Fig. 5, but organized by organ). Fig. S10 Principal components analysis of glycome profiling data (data are presented for all samples from the six fractions obtained during the sequential extraction). Fig. S11 Principal components analysis of glycome profiling data (data is presented for each individual extraction step performed during the sequential extraction). Fig. S12 Principal components analysis of glycome profiling (data presented independently for each organ). Table S1 Listing of plant cell wall glycan‐directed monoclonal antibodies (mAbs) used in the glycome profiling screening Table S2 Amount of [file NPH-213-1710-s001.pdf]

New Phytologist

***New Phytologist* Supporting Information**

Article title: **A cell wall reference profile for *Miscanthus* bioenergy-crops highlights compositional and structural variations associated with development and organ origin**

Authors: Ricardo M. F. da Costa, Sivakumar Pattathil, Utku Avci, Scott J. Lee, Samuel P. Hazen, Ana Winters, Michael G Hahn and Maurice Bosch

Article acceptance date: 29 September 2016

The following Supporting Information is available for this article:

Table S1 Listing of plant cell wall glycan-directed monoclonal antibodies (mAbs) used in the glycome profiling screening.

Fig. S1 Distribution of measurements of the minor monosaccharides fucose (Fuc) and Galactose (Gal) released upon acid hydrolysis of miscanthus CWM.

Fig. S2 Distribution of measurements of the major monosaccharides Arabinose (Ara), Glucose (Glc) and Xylose (Xyl) released upon acid hydrolysis of miscanthus CWM.

Fig. S3 Distribution of measurements of the arabinose to xylose ratio (Ara/Xyl) of miscanthus CWM.

Fig. S4 Distribution of measurements of released acetate upon 0.1 M KOH treatment of miscanthus CWM.

Fig. S5 Distribution of measurements of ferulic (FA) and *p*-coumaric (*p*CA) acid released upon 1 M KOH treatment of miscanthus CWM.

Fig. S6 Total carbohydrate recovered from each sequential extraction step per gram of purified cell-wall material (mg g^{-1} CWM) estimated by the phenol-sulphuric acid assay.

Table S2 Amount of carbohydrate recovered at each extraction step per gram of isolated cell wall material (mg g^{-1} CWM) based on phenol-sulphuric acid assay for total sugar estimation.

Table S3 Monosaccharide and acetyl bromide soluble lignin contents of the residue left after the sequential extraction.

Table S4 Pearson coefficients and associated probability (*P*) of the correlations between the results obtained for the *C. phytofermentans*-mediated digestibility assessment assay and several cell wall features.

Fig. S7 Heat map of the standard deviations (SD) from the mean binding intensities shown in Fig. 2.

Fig. S8 Immunofluorescent labelling of cell wall glycan epitopes in transverse sections from leaves and stems from *M.* × *giganteus* (gig01) with CCRC-M174 (galactomannan-2) and before a base-treatment with 0.1 M KOH for CCRC-M155 (xylan-5, Me-Glc substituted xylan).

Fig. S9 Mean binding values to different classes of cell wall glycan epitopes released at sequential extraction steps from leaf and stem samples from miscanthus biomass at 3 developmental stages (same data as in Fig. 5, but organised by organ).

Fig. S10 Principal components analysis of glycome profiling data (data is presented for all samples from the six fractions obtained during the sequential extraction).

Fig. S11 Principal components analysis of glycome profiling data (data is presented for each individual extraction step performed during the sequential extraction).

Fig. S12 Principal components analysis of glycome profiling (data presented independently for each organ).

Table S1

Listing of plant cell wall glycan-directed monoclonal antibodies (mAbs) used in the glycome profiling screening. The groupings of antibodies are based on a hierarchical clustering of ELISA data generated from a screen of all mAbs against a panel of plant polysaccharide preparations (Pattathil *et al.*, 2010), which grouped the mAbs according to the predominant polysaccharides recognised. Most listed items contain a web link to the WallMabDB plant cell wall monoclonal antibody database (http://www.wallmabdb.net), which provides detailed descriptions of each mAb, including immunogen, antibody isotype, epitope structure (to the current known extent), supplier information, and related literature citations.

Glycan Group Recognised	mAb Name
Non-Fucosylated Xyloglucan-1	CCRC-M95
	CCRC-M101
Non-Fucosylated Xyloglucan-2	CCRC-M104
	CCRC-M89
	CCRC-M93
	CCRC-M87
	CCRC-M88
Non-Fucosylated Xyloglucan-3	CCRC-M100
	CCRC-M103
Non-Fucosylated Xyloglucan-4	CCRC-M58
	CCRC-M86
	CCRC-M55
	CCRC-M52
	CCRC-M99
Non-Fucosylated Xyloglucan-5	CCRC-M54
	CCRC-M48
	CCRC-M49
	CCRC-M96
	CCRC-M50
	CCRC-M51
	CCRC-M53
Non-Fucosylated Xyloglucan-6	CCRC-M57
Fucosylated Xyloglucan	CCRC-M102
	CCRC-M39
	CCRC-M106
	CCRC-M84
	CCRC-M1
Xylan-1/XG	CCRC-M111
	CCRC-M108
	CCRC-M109
Xylan-2	CCRC-M119
	CCRC-M115
	CCRC-M110
	CCRC-M105
Xylan-3	CCRC-M117
	CCRC-M113
	CCRC-M120
	CCRC-M118
	CCRC-M116
	CCRC-M114
Xylan-4	CCRC-M154
	CCRC-M150

Glycan Group Recognised	mAbNames
Xylan-5	CCRC-M144
	CCRC-M146
	CCRC-M145
	CCRC-M155
Xylan-6	CCRC-M153
	CCRC-M151
	CCRC-M148
	CCRC-M140
	CCRC-M139
	CCRC-M138
Xylan-7	CCRC-M160
	CCRC-M137
	CCRC-M152
	CCRC-M149
Galactomannan-1	CCRC-M75
	CCRC-M70
	CCRC-M74
Galactomannan-2	CCRC-M166
	CCRC-M168
	CCRC-M174
	CCRC-M175
Glucomannan	CCRC-M169
	CCRC-M170
β-Glucan	LAMP
	BG1
HG Backbone-1	CCRC-M131
	CCRC-M38
	JIM5
HG Backbone-2	JIM136
	JIM7
RG-I Backbone	CCRC-M69
	CCRC-M35
	CCRC-M36
	CCRC-M14
	CCRC-M129
	CCRC-M72
Linseed Mucilage RG-I	JIM3
	CCRC-M40
	CCRC-M161
	CCRC-M164
Physcomitrella Pectin	CCRC-M98
	CCRC-M94
RG-Ia	CCRC-M5
	CCRC-M2
RG-Ib	JIM137
	JIM101
	CCRC-M61
	CCRC-M30
RG-Ic	CCRC-M23
	CCRC-M17
	CCRC-M19
	CCRC-M18
	CCRC-M56
	CCRC-M16

Glycan Group Recognised	mAbNames
RG-I/Arabinogalactan	CCRC-M60
	CCRC-M41
	CCRC-M80
	CCRC-M79
	CCRC-M44
	CCRC-M33
	CCRC-M32
	CCRC-M13
	CCRC-M42
	CCRC-M24
	CCRC-M12
	CCRC-M7
	CCRC-M77
	CCRC-M25
	CCRC-M9
	CCRC-M128
	CCRC-M126
	CCRC-M134
	CCRC-M125
	CCRC-M123
	CCRC-M122
	CCRC-M121
	CCRC-M112
	CCRC-M21
	JIM131
	CCRC-M22
	JIM132
	JIM1
	CCRC-M15
	CCRC-M8
	JIM16
Arabinogalactan-1	JIM93
	JIM94
	JIM11
	MAC204
	JIM20
Arabinogalactan-2	JIM14
	JIM19
	JIM12
	CCRC-M133
	CCRC-M107
Arabinogalactan-3	JIM4
	CCRC-M31
	JIM17
	CCRC-M26
	JIM15
	JIM8
	CCRC-M85
	CCRC-M81
	MAC266
	PN16.4B4
Arabinogalactan-4	MAC207
	JIM133
	JIM13
	CCRC-M92
	CCRC-M91
	CCRC-M78
Unidentified	MAC265
	CCRC-M97

Fig. S1

Distribution of measurements of the minor monosaccharides fucose (Fuc) and Galactose (Gal) released upon acid hydrolysis of miscanthus CWM. Values are expressed as percentage of cell wall material dry weight (% CWM) from leaf and stem for 8 miscanthus genotypes examined in this study (*M.* × *giganteus*: gig01; *M. sinensis*: sin08, sin09, sin11, sin13, sin15; *M. sacchariflorus*: sac01; this holds true for the remaining figures where the 8 genotypes are mentioned; for more details, please consult the Materials and Methods section). Developmental stages: active growth (AGr), peak biomass (PBm) and senescence (SSn). The non-outlier range is defined as the range of values which fall outside 1.5× the interquartile range of the distribution (height of the 25% – 75% box). Not significantly different developmental stages are indicated by a common underlined letter next to the box (Tukey's test at α=0.05).

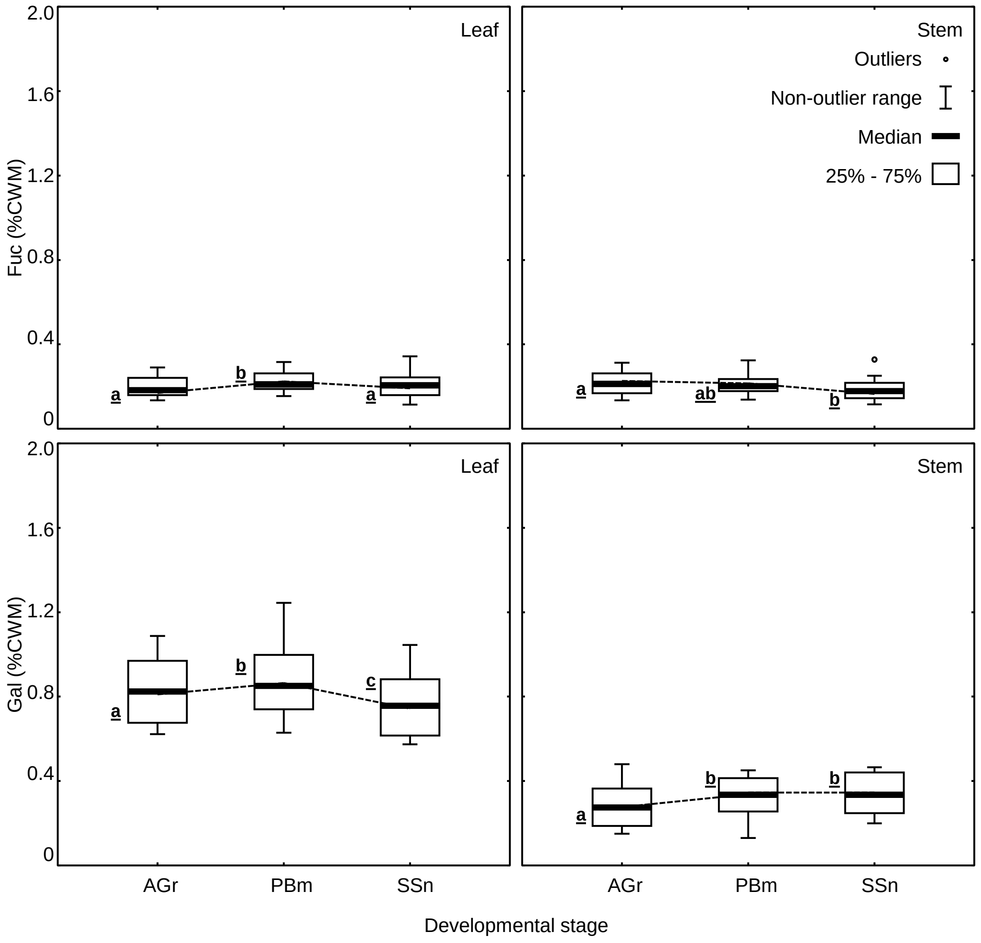

Fig. S2

Distribution of measurements of the major monosaccharides Arabinose (Ara), Glucose (Glc) and Xylose (Xyl) released upon acid hydrolysis of miscanthus CWM. Values are expressed as percentage of cell wall material dry weight (% CWM) from leaf and stem for 8 miscanthus genotypes at active growth (AGr), peak biomass (PBm) and senescence (SSn). The non-outlier range is defined as the range of values which fall outside 1.5× the interquartile range of the distribution (height of the 25% – 75% box). Not significantly different developmental stages are indicated by a common underlined letter next to the box (Tukey's test at α=0.05).

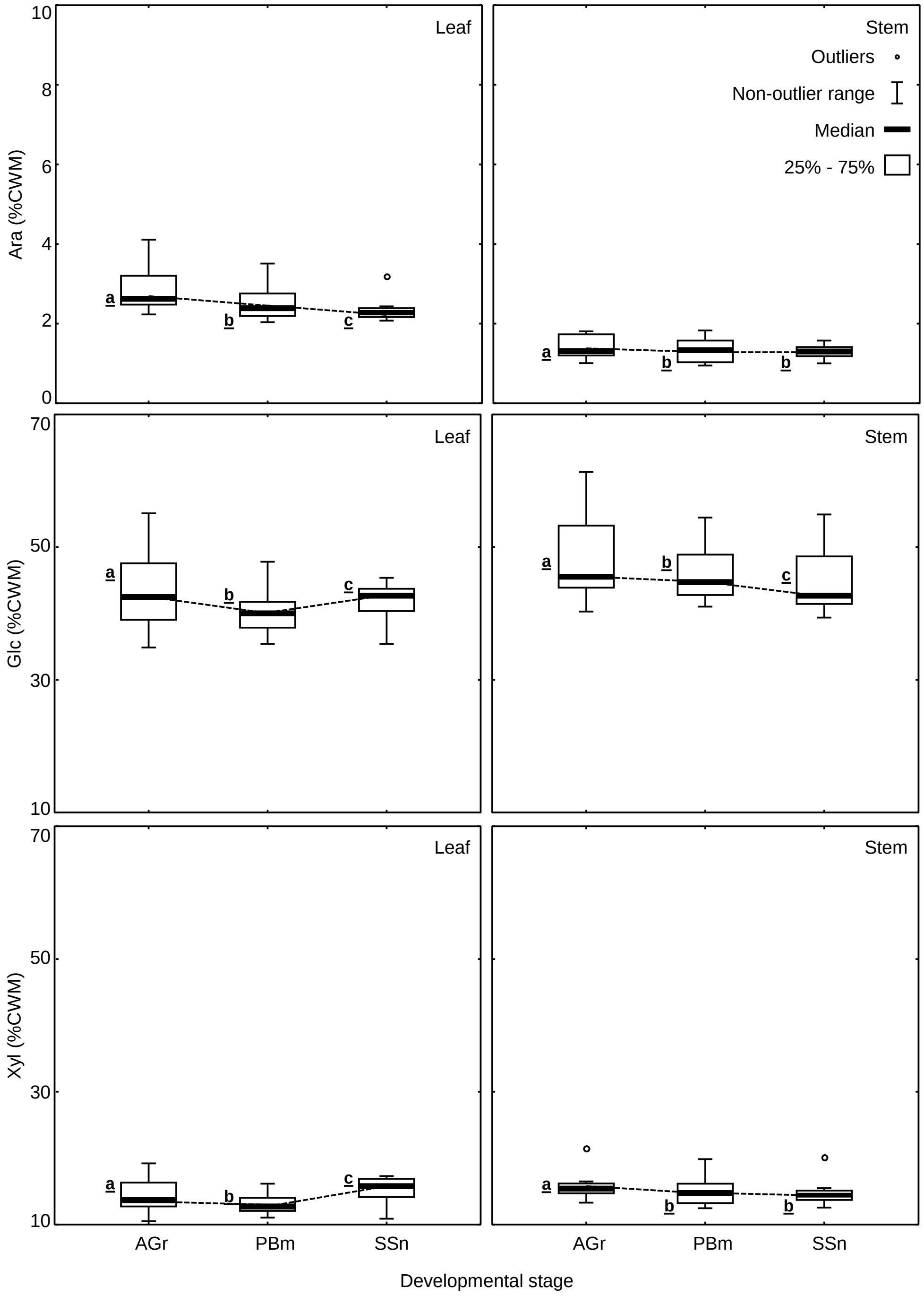

Fig. S3

Distribution of measurements of the arabinose to xylose ratio (Ara/Xyl) of miscanthus CWM. Values were calculated by dividing the percentage of Ara by the percentage of Xyl in each cell wall sample from leaf and stem of all miscanthus genotypes at active growth (AGr), peak biomass (PBm) and senescence (SSn). The non-outlier range is defined as the range of values which fall outside 1.5× the interquartile range of the distribution (height of the 25% – 75% box). Not significantly different developmental stages are indicated by a common underlined letter next to the box (Tukey's test at α=0.05).

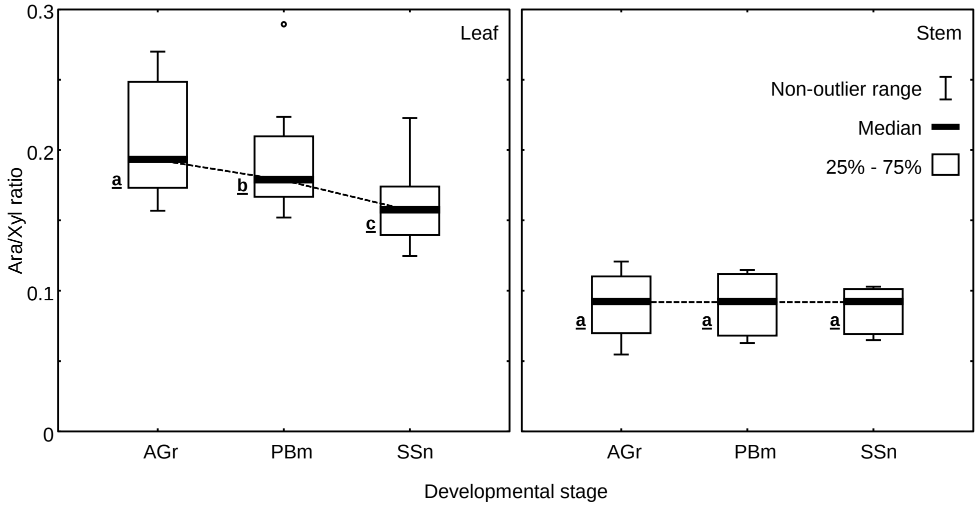

Fig. S4

Distribution of measurements of released acetate upon 0.1 M KOH treatment of miscanthus CWM. Values are expressed as percentage of cell wall material dry weight (% CWM) from leaf and stem for all miscanthus genotypes at active growth (AGr), peak biomass (PBm) and senescence (SSn). The non-outlier range is defined as the range of values which fall outside 1.5× the interquartile range of the distribution (height of the 25% – 75% box). Not significantly different developmental stages are indicated by a common underlined letter next to the box (Tukey's test at α=0.05).

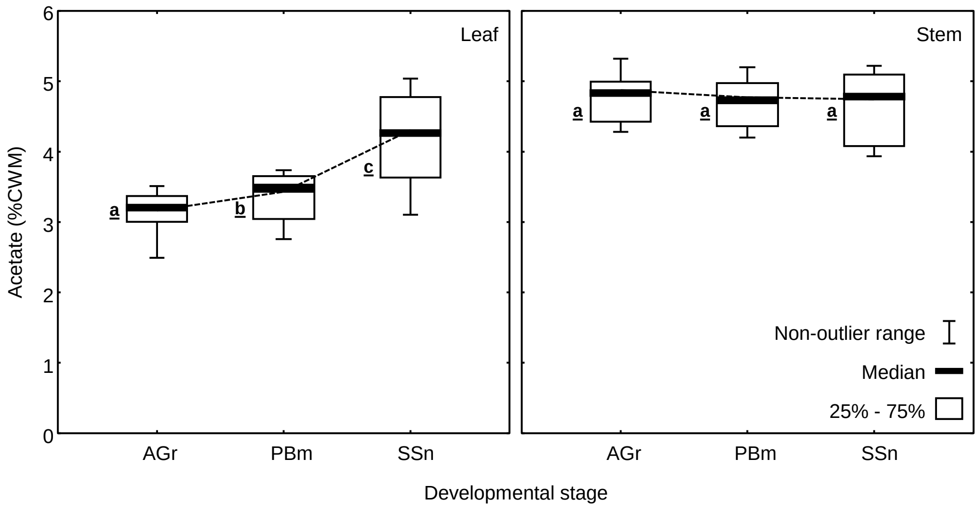

Fig. S5

Distribution of measurements of ferulic (FA) and *p*-coumaric (*p*CA) acid released upon 1 M KOH treatment of miscanthus CWM. Values are expressed as percentage of cell wall material dry weight (% CWM) from leaf and stem for all miscanthus genotypes at active growth (AGr), peak biomass (PBm) and senescence (SSn). The non-outlier range is defined as the range of values which fall outside 1.5× the interquartile range of the distribution (height of the 25% – 75% box). Not significantly different developmental stages are indicated by a common underlined letter next to the box (Tukey's test at α=0.05).

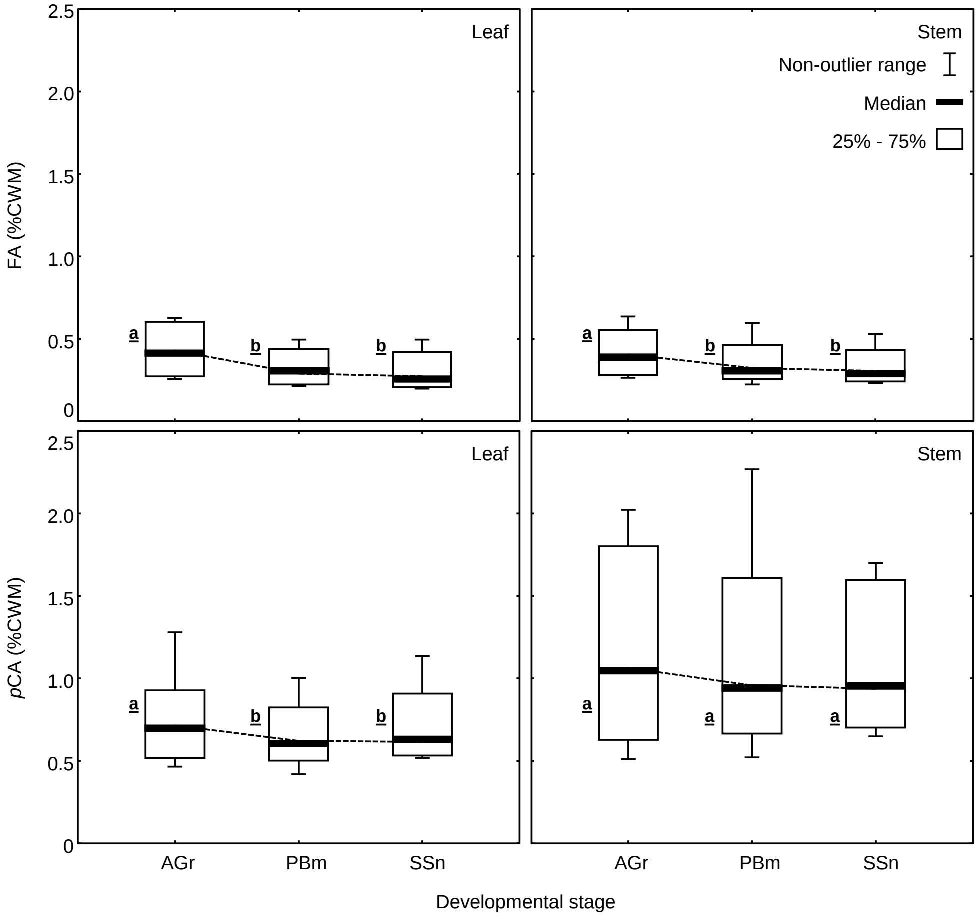

New Phytologist

Fig. S6

Total carbohydrate recovered from each sequential extraction step per gram of purified cell wall material (mg g^{-1} CWM) estimated by the phenol-sulphuric acid assay. Values are the mean ± SD of all genotypes. Abbreviations: AGr, actively growing; PBm, peak biomass; SSn, senescence.

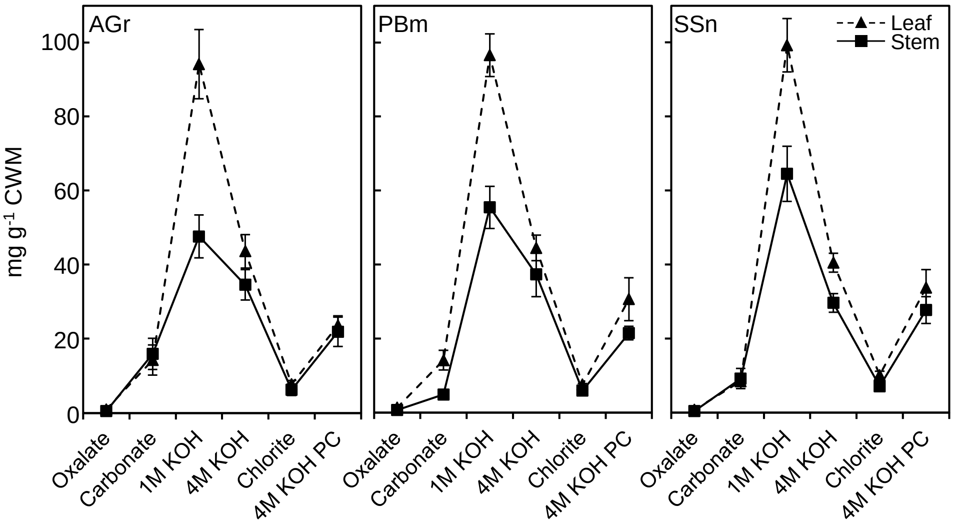

Table S2

Amount of carbohydrate recovered at each extraction step per gram of isolated cell wall material (mg Glc equivalent g^{-1} CWM) based on phenol-sulphuric acid assay for total sugar estimation using glucose as a standard. (AGr: actively growing; PBm: peak biomass; SSn: senescence).

**Ammonium Oxalate**					
0.81					
**Leaf**			**Stem**		
1.04			0.57		
**AGr**	**PBm**	**SSn**	**AGr**	**PBm**	**SSn**
0.99	1.36	0.78	0.55	0.73	0.44

**Carbonate**					
11.19					
**Leaf**			**Stem**		
12.35			10.03		
**AGr**	**PBm**	**SSn**	**AGr**	**PBm**	**SSn**
14.34	14.21	8.52	15.97	4.91	9.22

**1M KOH**					
76.35					
**Leaf**			**Stem**		
96.76			55.93		
**AGr**	**PBm**	**SSn**	**AGr**	**PBm**	**SSn**
94.28	96.65	99.36	47.69	55.52	64.57

**4M KOH**					
38.42					
**Leaf**			**Stem**		
42.94			33.91		
**AGr**	**PBm**	**SSn**	**AGr**	**PBm**	**SSn**
43.70	44.55	40.56	34.64	37.40	29.68

**Chlorite**					
7.48					
**Leaf**			**Stem**		
8.51			6.45		
**AGr**	**PBm**	**SSn**	**AGr**	**PBm**	**SSn**
7.78	7.62	10.14	6.27	5.94	7.12

**4M KOH PC**					
26.57					
**Leaf**			**Stem**		
29.39			23.75		
**AGr**	**PBm**	**SSn**	**AGr**	**PBm**	**SSn**
23.68	30.69	33.79	21.96	21.52	27.76

Table S3

Monosaccharide and **a**cetyl bromide soluble lignin contents of the residue left after the sequential extraction done as part of the glycome profiling method. Values are expressed as percentage (%) of the dry weight of the residue remaining from leaf or stem CWM samples at three developmental stages and are the mean ± SD of all studied miscanthus genotypes. Other constituents of the residues may include salts of the chemicals used during the sequential extraction, and water, as a result of the hygroscopic effect of KOH.

		Fucose	Arabinose	Galactose	Glucose	Xylose	Lignin
**Active Growth**	**Leaf**	<0.01	<0.01	<0.01	84.41 ± 9.78	1.21 ± 0.80	0.73 ± 0.14
	**Stem**	<0.01	<0.01	<0.01	83.56 ± 11.86	1.90 ± 0.93	1.02 ± 0.31
**Peak Biomass**	**Leaf**	<0.01	<0.01	<0.01	85.45 ± 8.02	1.23 ± 0.64	0.86 ± 0.26
	**Stem**	<0.01	<0.01	<0.01	86.93 ± 6.14	1.84 ± 0.58	1.17 ± 0.30
**Senescence**	**Leaf**	<0.01	<0.01	<0.01	81.32 ± 9.33	1.42 ± 0.45	0.94 ± 0.21
	**Stem**	<0.01	<0.01	<0.01	88.29 ± 5.89	2.00 ± 0.60	1.24 ± 0.30

Table S4

Pearson coefficients and associated probability (*P*) of the correlations between the results obtained for the *C. phytofermentans*-mediated digestibility assessment assay (mg of ethanol yielded per g of dry cell wall material, CWM) and several cell wall features (% CWM). For each correlation coefficient, *N* = 96 for all samples, *N* = 32 for individual developmental stages, and N = 48 for individual organs. Highlighted correlations are significant at *P*<0.05. Abbreviations: Ara/Xyl, ratio between arabinose and xylose content; FA, ester-linked ferulic acid; *p*CA, ester-linked *p*-coumaric acid.

	**Glucose**	**Xylose**	**Ara/Xyl**	**FA**	***p*CA**	**Acetate**	**Lignin**
**All**	-0.0406 *P*=0.694	**-0.2159** ***P*=0.035**	**0.2595** ***P*=0.011**	**0.1868** ***P*=0.068**	-0.0786 *P*=0.446	**-0.2891** ***P*=0.004**	**-0.5914** ***P*<0.001**
**Leaf**	0.0627 *P*=0.672	-0.1793 *P*=0.223	**0.3219** ***P*=0.026**	**0.3457** ***P*=0.016**	0.0698 *P*=0.637	**-0.5193** ***P*<0.001**	**-0.5545** ***P*<0.001**
**Stem**	-0.0518 *P*=0.726	-0.2149 *P*=0.142	0.175 *P*=0.234	-0.0409 *P*=0.782	-0.0957 *P*=0.517	0.1963 *P*=0.181	**-0.6515** ***P*<0.001**

Fig. S7A

Standard deviations (SD) from the mean binding intensities shown in Fig. 2. Cell wall glycan classes are indicated to the right, and corresponding extract, organ and developmental stage are labelled below each column. SD values are given in optical density (OD) units and the maximum value is 0.4755 (≈0.5). A different colour palette is here used to avoid confusion with the heat maps of the means presented in Fig. 2.

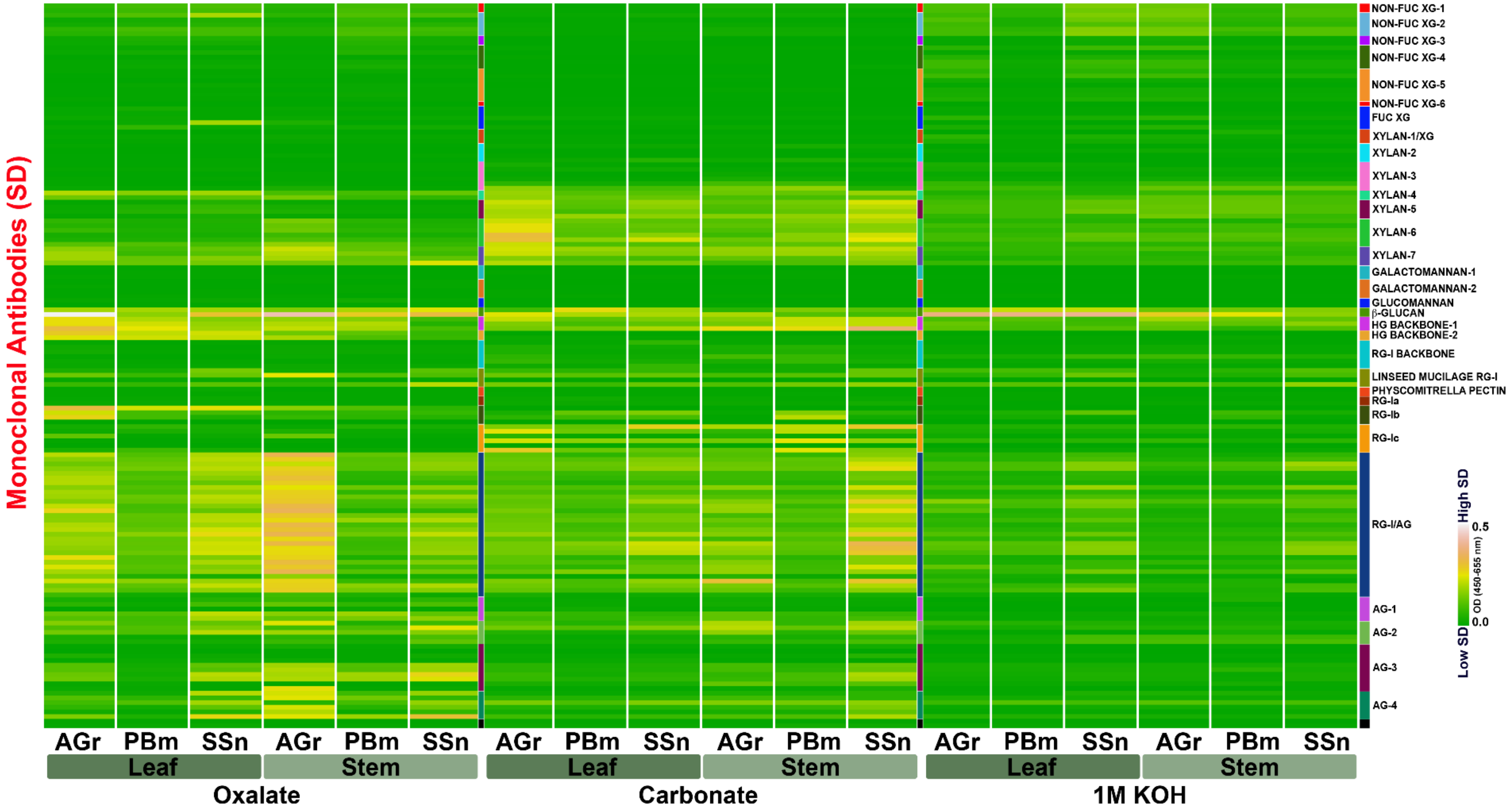

Fig. S7B

Standard deviations (SD) from the mean binding intensities shown in Fig. 2. Cell wall glycan classes are indicated to the right, and corresponding extract, organ and developmental stage are labelled below each column. SD values are given in optical density (OD) units and the maximum value is 0.4755 (≈0.5). A different colour palette is here used to avoid confusion with the heat maps of the means presented in Fig. 2.

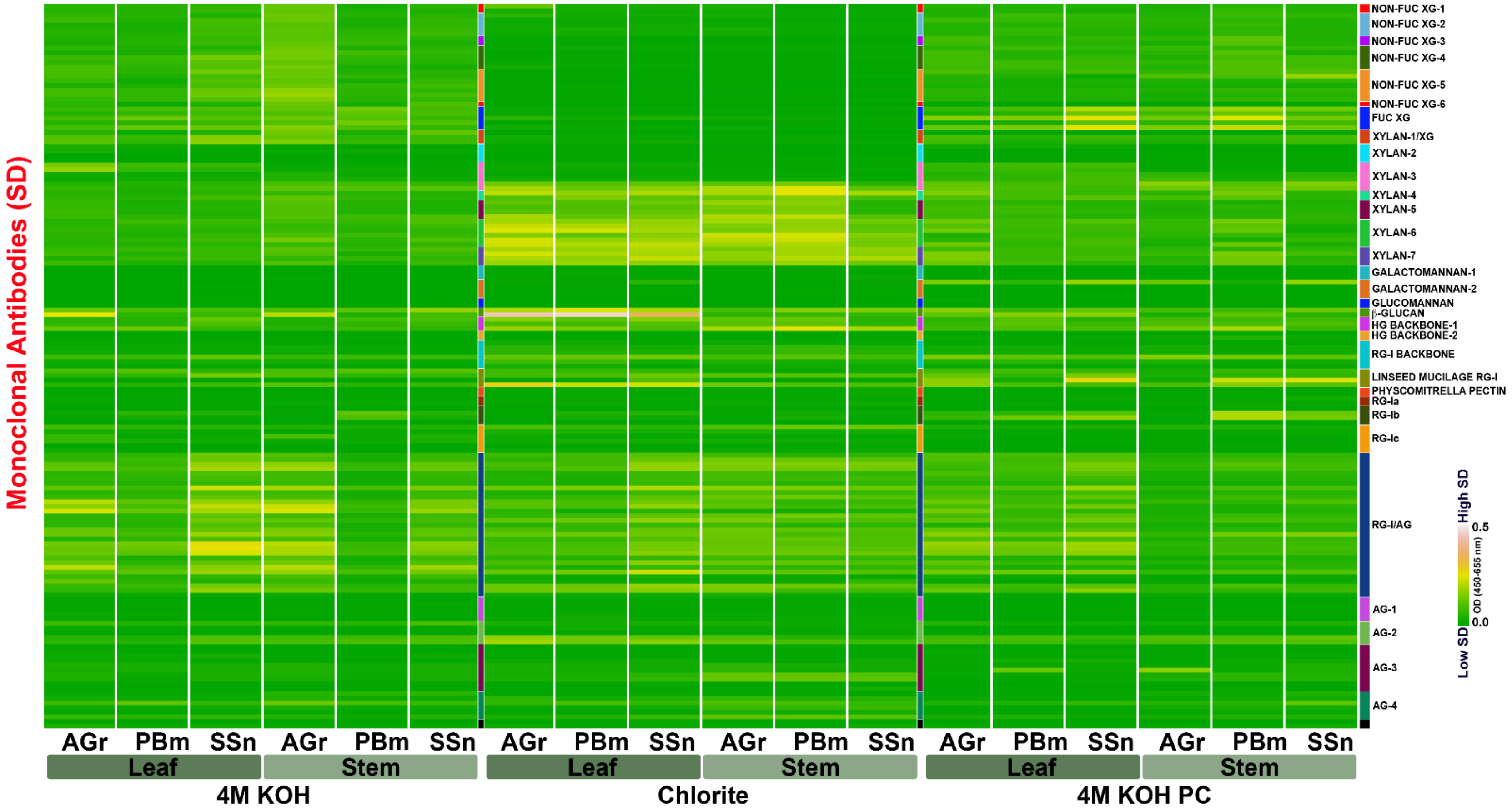

Fig. S8

Immunofluorescent labelling of cell wall glycan epitopes in untreated transverse sections from leaves and stems from *M.* × *giganteus* (gig01) with CCRC-M174 (galactomannan-2) and for CCRC-M155 (xylan-5). Low or negligible binding signals to glycan epitopes observed in these sections serve as negative controls to the results presented in Fig. 3. Bars, 100 µm. *In situ* immunolabelling studies with a larger set of mAbs has been performed, but not reported here. More information is available upon request.

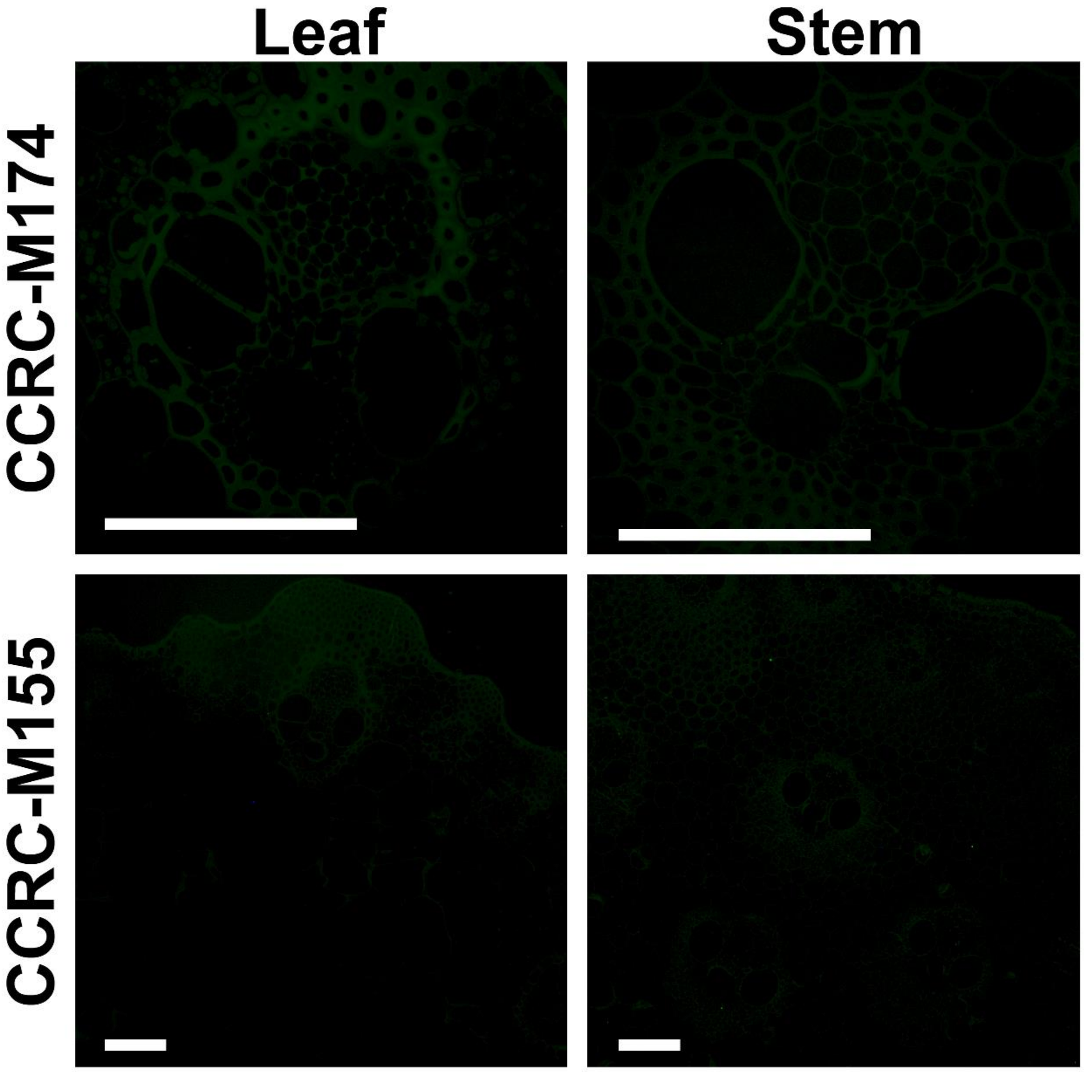

Fig. S9

Mean binding values to different classes of cell wall glycan epitopes released at sequential extraction steps from leaf and stem samples from miscanthus biomass at 3 developmental stages (same data as in Fig. 5, but organised by organ).

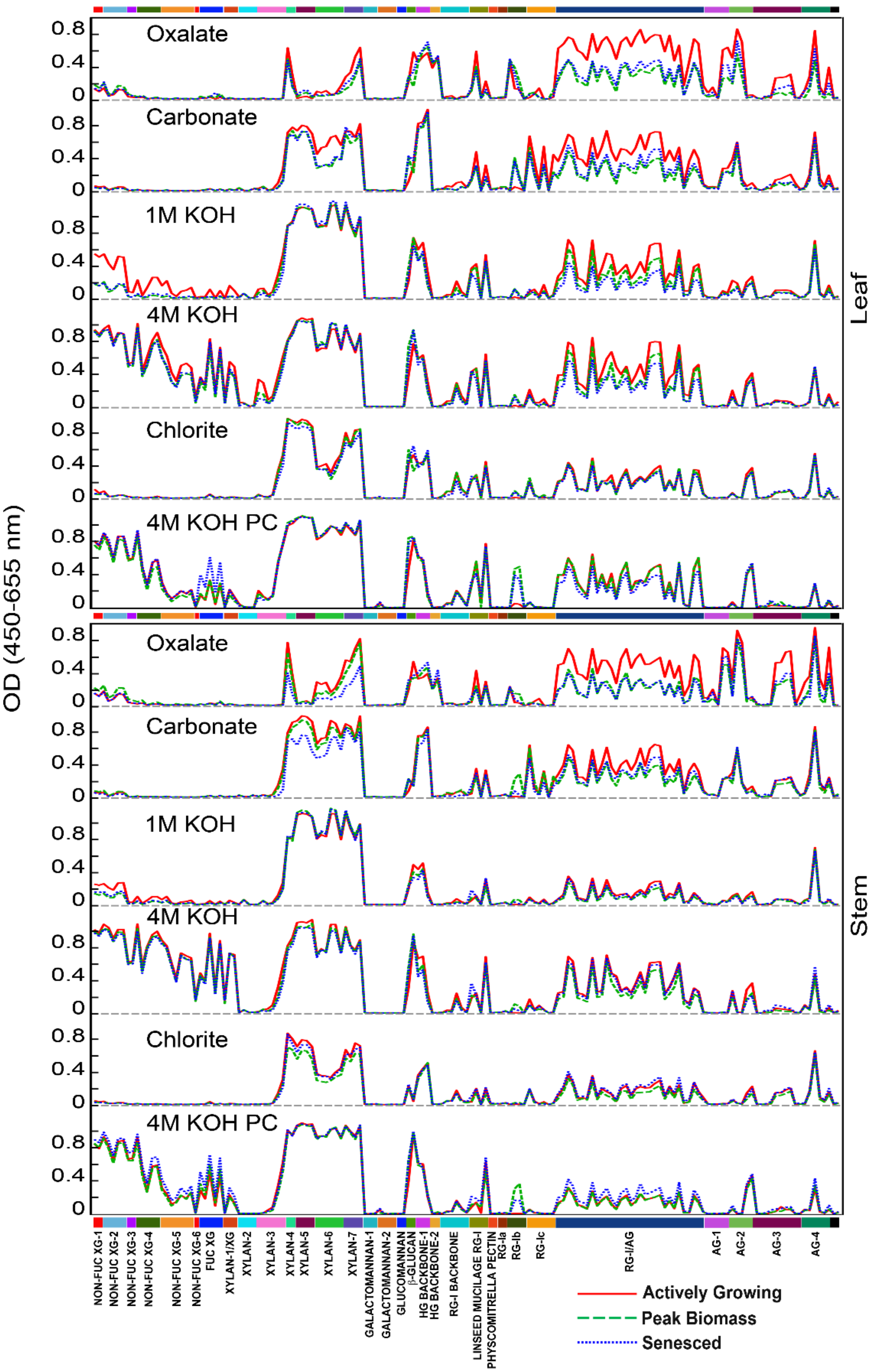
Oxalate
Carbonate
1M KOH
4M KOH
Chlorite
4M KOH PC
0.8
0.4
0
OD (450-655 nm)
Leaf
Stem
NON-FUC XG-1
NON-FUC XG-2
NON-FUC XG-3
NON-FUC XG-4
NON-FUC XG-5
NON-FUC XG-6
FUC XG
XYLAN-1/XG
XYLAN-2
XYLAN-3
XYLAN-4
XYLAN-5
XYLAN-6
XYLAN-7
GALACTOMANNAN-1
GALACTOMANNAN-2
GLUCOMANNAN
β-GLUCAN
HG BACKBONE-1
HG BACKBONE-2
RG-I BACKBONE
LINSEED MUCILAGE RG-I
PHYSCOMITRELLA PECTIN
RG-Ia
RG-Ib
RG-Ic
RG-I/AG
AG-1
AG-2
AG-3
AG-4
Actively Growing
Peak Biomass
Senesced

Fig. S10

Principal components analysis of glycome profiling data. Plot of principal component one (PC1) and principal component two (PC2) scores and corresponding loadings plots of the PC along which clusters emerged. Data are presented for all samples from the six fractions obtained during the sequential extraction.

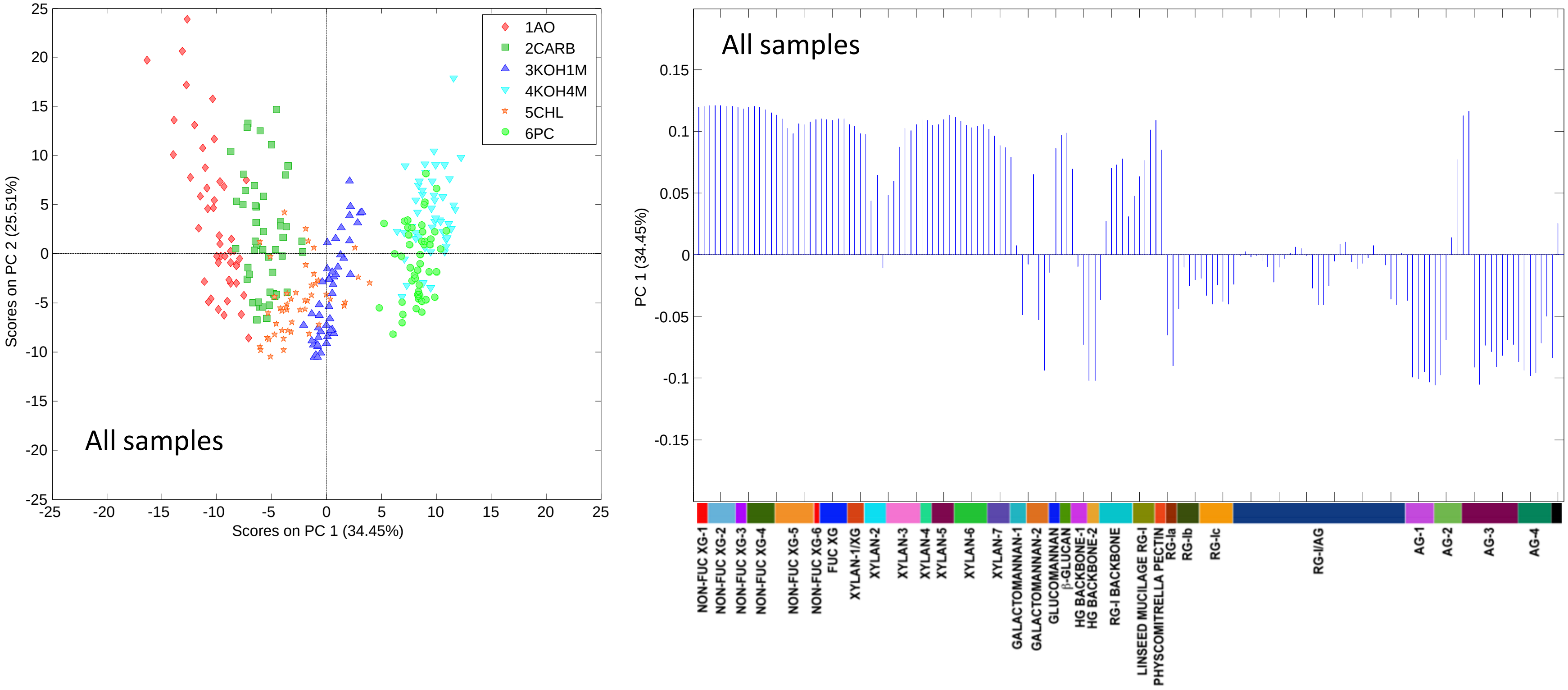

Fig. S11

Principal components analysis of glycome profiling data. Plot of principal component one (PC1) and principal component two (PC2) scores and corresponding loadings plots of the PC along which clusters emerged. Data are presented for each individual extraction step performed during the sequential extraction. Abbreviations: L, leaf samples; S, stem samples.

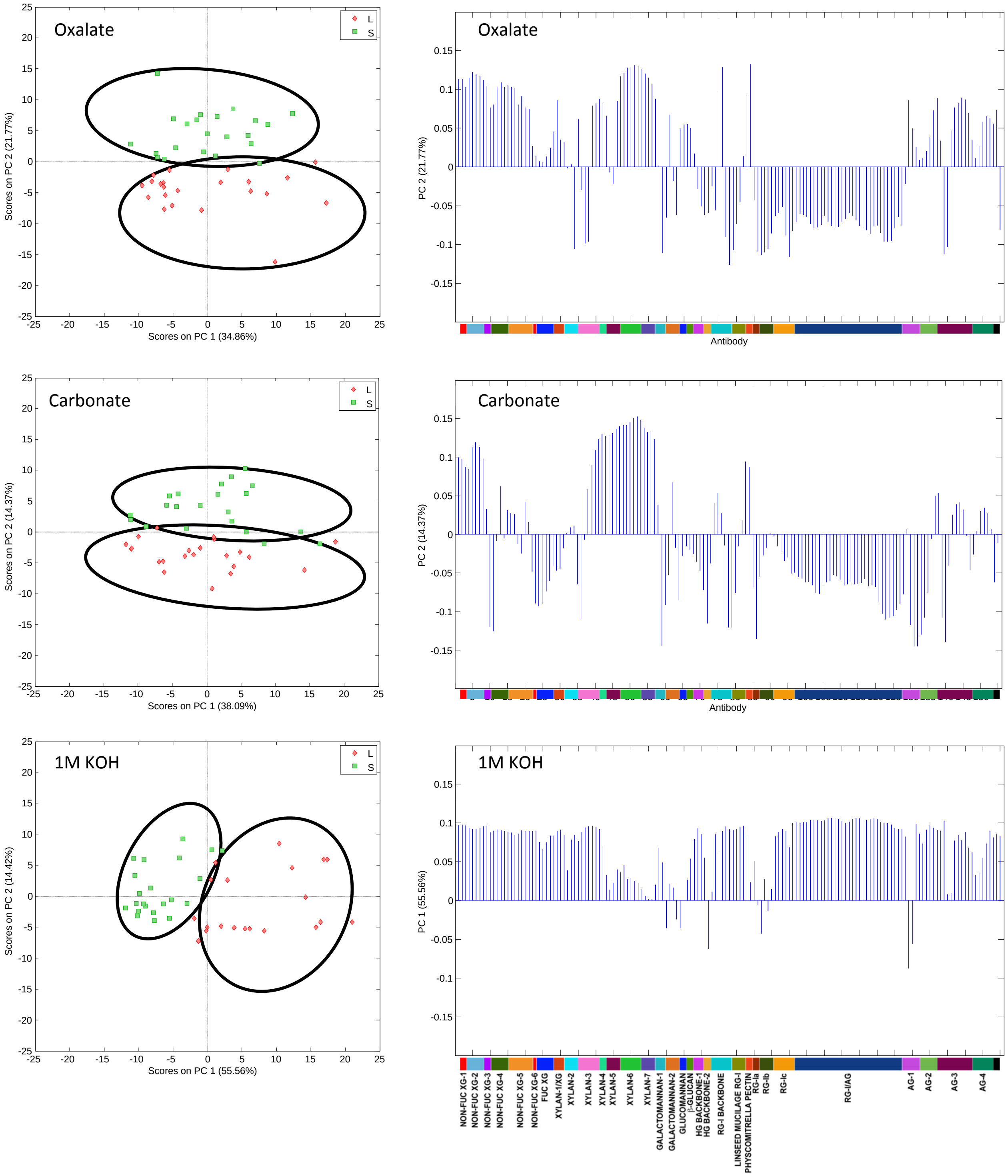

New Phytologist

Fig. S11 (continued)

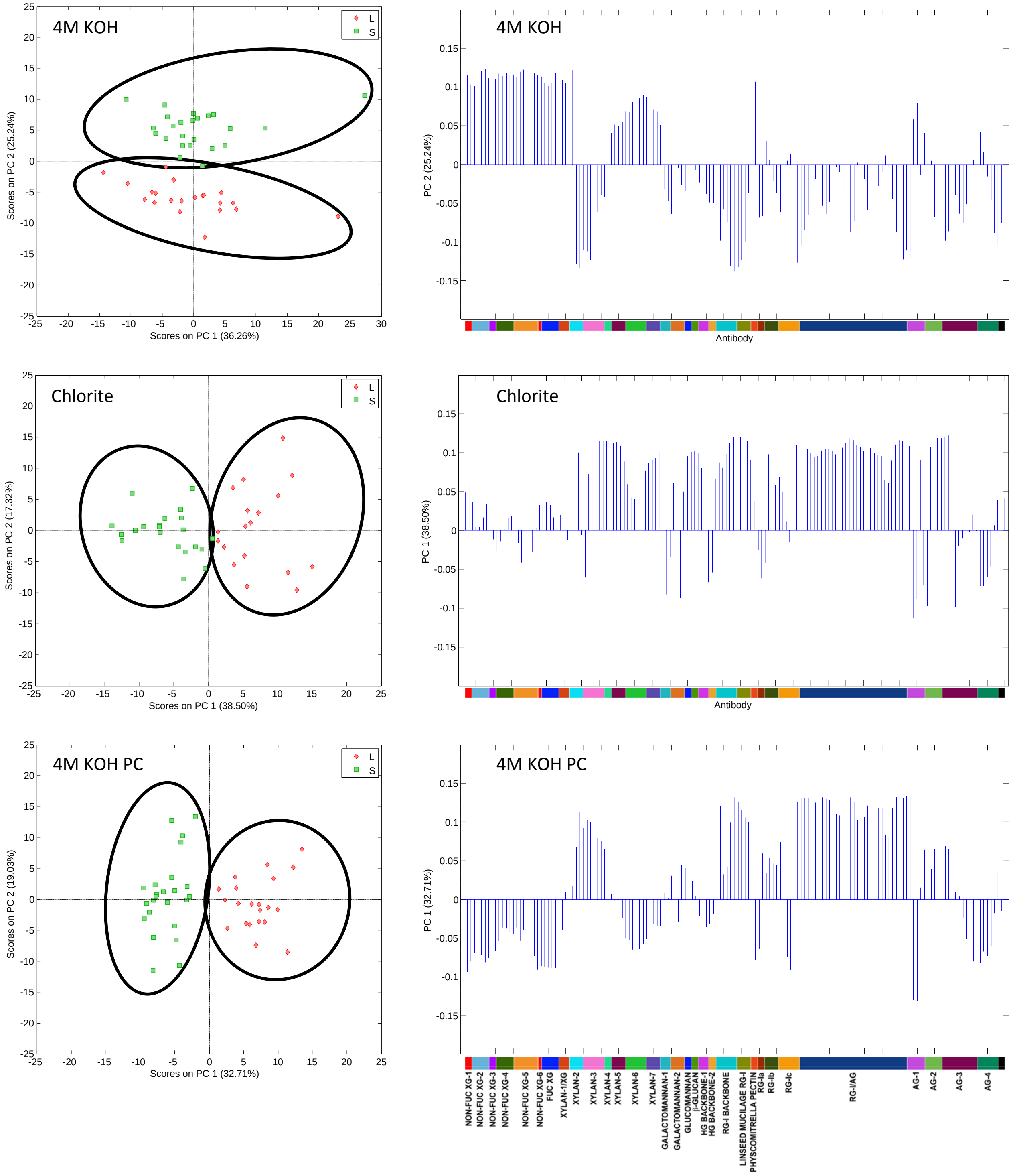

Fig. S12

Principal components analysis of glycome profiling data. Plot of principal component one (PC1) and principal component two (PC2) scores for each individual extraction step performed during the sequential extraction presented independently for each organ: stem samples on the left panel and leaf on the right. Corresponding loadings plots are presented for the PC analyses where clusters emerged. Abbreviations: AGr, active growth; PBm, peak biomass; SSn, senesced stage.

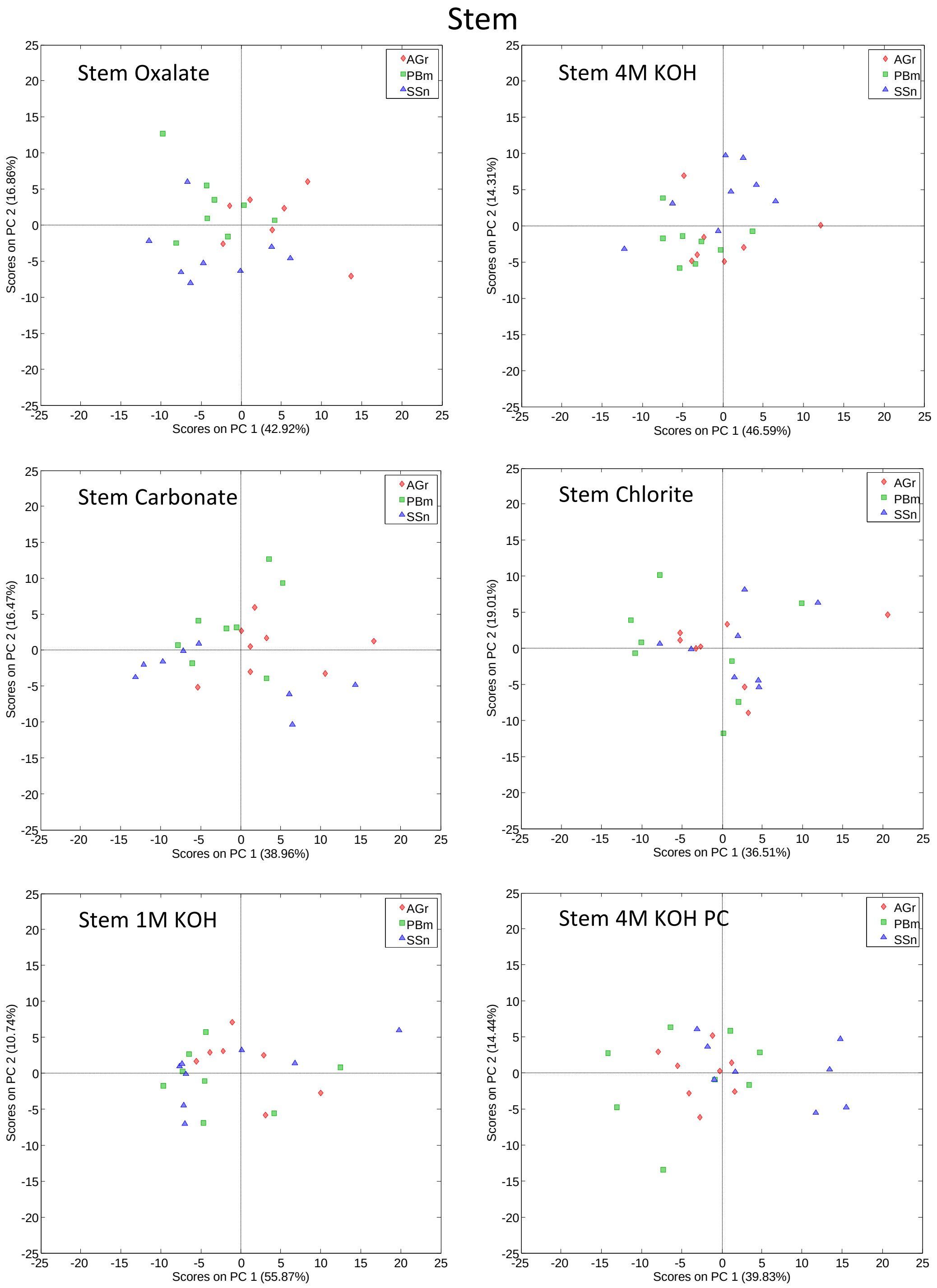

Fig. S12 (continued)

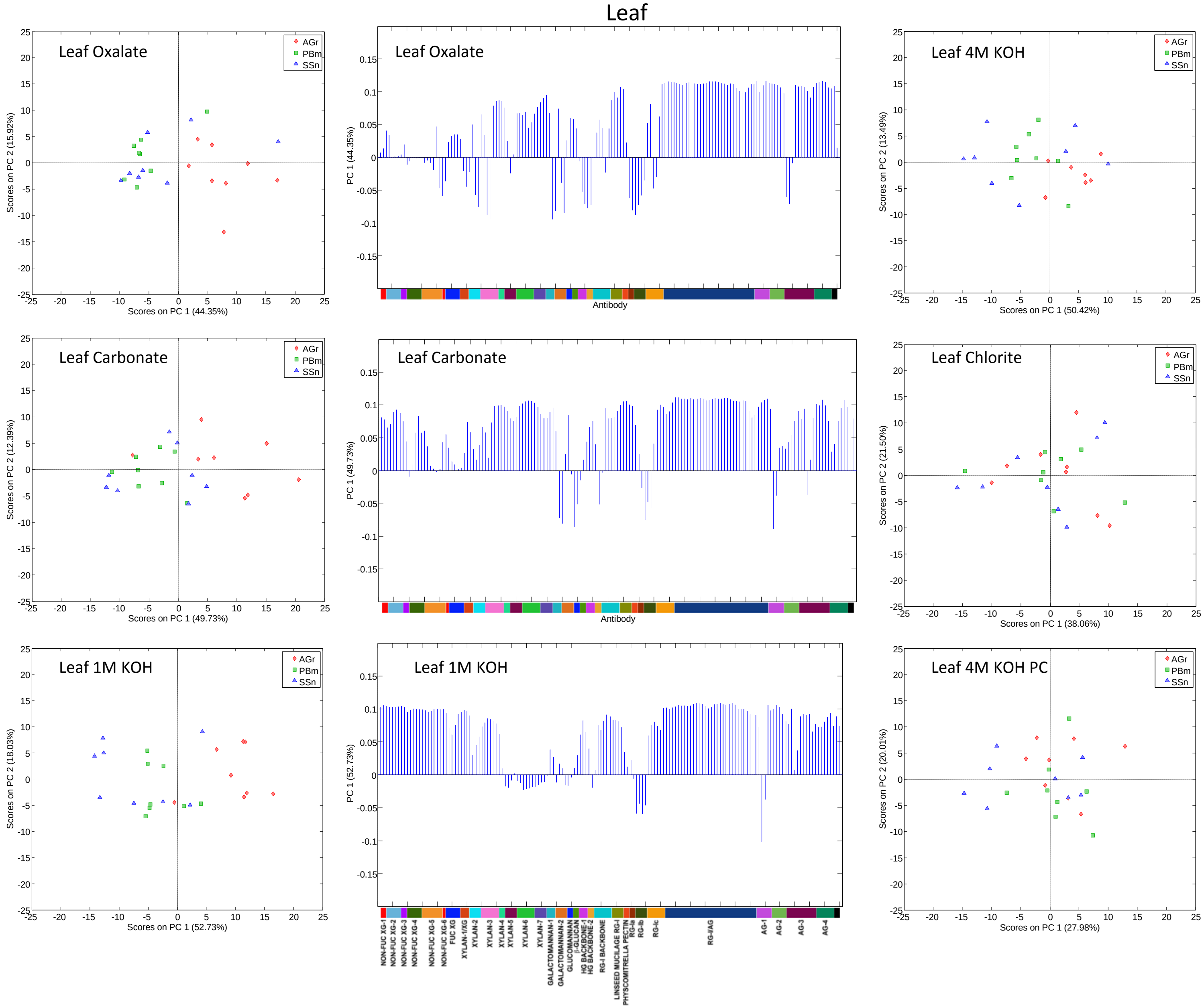

References

Pattathil S, Avci U, Baldwin D, Swennes AG, McGill JA, Popper Z, Bootten T, Albert A, Davis RH, Chennareddy C *et al.* 2010. A comprehensive toolkit of plant cell wall glycan-directed monoclonal antibodies. *Plant Physiology* **153**: 514–525.
